# Antisense Oligonucleotide-Mediated Transcript Knockdown in Zebrafish

**DOI:** 10.1371/journal.pone.0139504

**Published:** 2015-10-05

**Authors:** Andrea Pauli, Tessa G. Montague, Kim A. Lennox, Mark A. Behlke, Alexander F. Schier

**Affiliations:** 1 Department of Molecular and Cellular Biology, Harvard University, Cambridge, Masschusetts, United States of America; 2 Integrated DNA Technologies, Inc., Coralville, Iowa, United States of America; University Zürich, SWITZERLAND

## Abstract

Antisense oligonucleotides (ASOs) are synthetic, single-strand RNA-DNA hybrids that induce catalytic degradation of complementary cellular RNAs via RNase H. ASOs are widely used as gene knockdown reagents in tissue culture and in *Xenopus* and mouse model systems. To test their effectiveness in zebrafish, we targeted 20 developmental genes and compared the morphological changes with mutant and morpholino (MO)-induced phenotypes. ASO-mediated transcript knockdown reproduced the published loss-of-function phenotypes for *oep*, *chordin*, *dnd*, *ctnnb2*, *bmp7a*, *alk8*, *smad2* and *smad5* in a dosage-sensitive manner. ASOs knocked down both maternal and zygotic transcripts, as well as the long noncoding RNA (lncRNA) *MALAT1*. ASOs were only effective within a narrow concentration range and were toxic at higher concentrations. Despite this drawback, quantitation of knockdown efficiency and the ability to degrade lncRNAs make ASOs a useful knockdown reagent in zebrafish.

## Introduction

**Fig 1:**
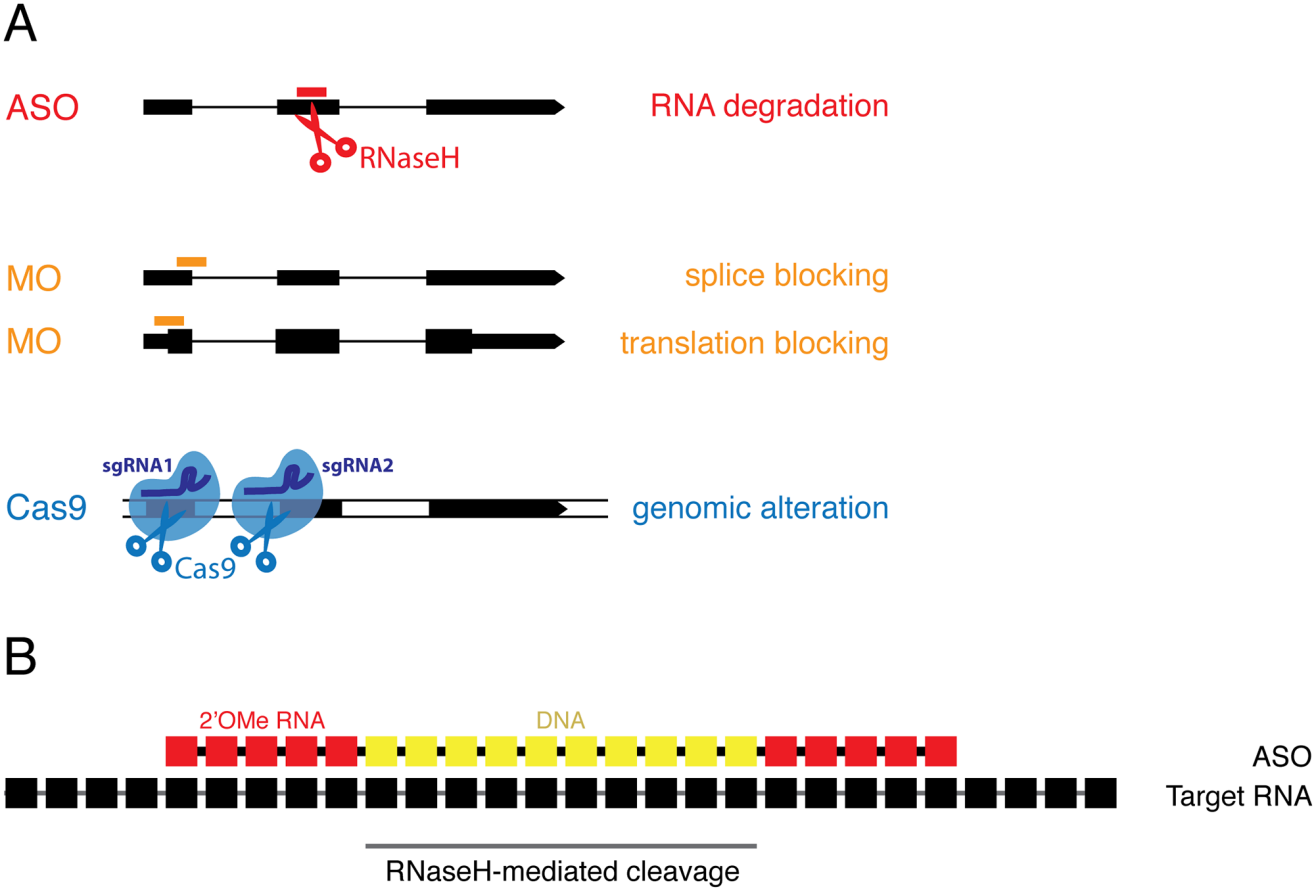
Overview of knockdown and loss-of-function technologies in zebrafish. A) Antisense oligos (ASOs, red) degrade target RNA, morpholinos (MOs, orange) either block splicing or inhibit translation, and Cas9-sgRNA complexes (blue) create double-strand breaks in DNA leading to genomic alterations. B) ASOs are RNA-DNA hybrid oligonucleotides containing 10 central DNA nucleotides flanked by 5 2’O-Methyl (2’OMe) modified RNA nucleotides on either side (5-10-5 arrangement). Individual nucleotides in the ASO are linked by phosphorothioate bonds to increase stability.

One effective strategy for interrogating gene function is to disrupt the generation of a gene product by knockdown or knockout. Knockout technologies, such as CRISPR/Cas9 and homologous recombination, alter the DNA locus of the gene by either introducing a premature stop codon or removing the entire locus (Fig 1A) [1,2]. Knockdown methods, on the other hand, such as RNAi, siRNAs and modified antisense oligonucleotides [3,4], target the mRNA rather than alter the DNA. While it is most reliable to infer gene function by generating a mutant organism, knockdown reagents can provide a more immediate assessment of gene function and can be used to target gene products without disrupting regulatory DNA elements.

Because siRNAs have been used with limited success in zebrafish [5], an alternative knockdown reagent, morpholino oligonucleotides (MOs), has been the preferred zebrafish knockdown reagent [6] (Fig 1A). MOs are synthetic oligonucleotides composed of around 25 nucleotides that possess a morpholine ring instead of the ribose ring found in DNA and RNA, with non-ionic phosphorodiamidate linkages instead of a phosphodiester backbone. This provides nuclease resistance and allows them to bind RNA molecules through conventional Watson-Crick base pairing. MOs interfere with gene function by either sterically hindering translation [7] or splicing [8] or functioning as target protectors [9] (Fig 1A). However, they do not degrade the target RNA, impeding quantitation of knockdown efficiency. This is particularly an issue for translation-blocking RNAs, which do not create changes to the splicing pattern of the target RNA. MOs can also cause significant off-target activity, resulting in misleading phenotypic artifacts [10]. An increasing number of mutants that have been generated subsequent to MO-based studies are either non-phenotypic or produce alternative phenotypes from the published MO-mediated knockdowns [11–14]. For this reason, the availability of an alternative knockdown reagent would be of use to complement MO studies.

The present study employs RNA-DNA hybrid ASOs (also known as gapmers), which are chimeric oligonucleotides containing 10 DNA nucleotides flanked by 5 2’O-Methyl (2’OMe) RNA residues (5-10-5 arrangement) [15,16]. The phosphate backbone in the DNA and RNA is replaced with phosphorothioate bonds to increase ASO stability, and addition of 2’OMe RNA modifications increases nuclease resistance (Fig 1B). The RNA and DNA portions in the hybrid molecule serve distinct functions: the RNA nucleotides increase affinity to complementary RNAs, while the central DNA stretch serves as a guide for RNase H-mediated degradation of the complementary RNA. This strategy has been widely used as a knockdown approach in *Xenopus* [17–20], tissue culture [21], mouse models (where gapmer ASOs have reversed disease phenotypes [22,23]), and it has shown promise in gene therapy [24,25]. RNase H degradation is catalytic, and the ASO itself is recycled, meaning that a single ASO can direct degradation of multiple copies of the target RNA. In contrast, a single steric-blocking MO can only bind and inactivate a single target RNA molecule.

ASOs offer a number of advantages over MOs. First, they cause degradation of the transcript via RNase H. Thus, the efficiency of the knockdown can be quantified. Second, due to degradation of the RNA, rather than prevention of splicing or translation, they can be used to eliminate spliced maternal RNAs. Third, they can target both protein-coding and noncoding RNAs due to activity in the nucleus: ASOs have been shown to shuttle between the cytoplasm and nucleoplasm [26], and can efficiently target nuclear-retained long noncoding RNAs (lncRNAs) [22,27] and nascent RNAs [16]. Finally, ASOs are significantly cheaper than MOs, with an average current cost (as of July 2015) of ~$200 (rather than ~$400). Additionally, only 1/10-1/100 of the MO concentration is required for ASO experiments. Therefore, ASOs combine several properties (quantifiable knockdown rates, specificity, efficiency, nuclear activity and persistence *in vivo* [22]) that highlight their potential as alternatives to MO-mediated knockdown.

To test the feasibility of using ASOs as an alternative knockdown reagent in zebrafish, we targeted 18 genes with known embryonic loss-of-function phenotypes. ASO-mediated knockdown reproduced the published phenotypes for 8 developmental protein-coding genes (*one-eyed pinhead* (*oep)*, *chordin*, *dead-end* (*dnd)*, *beta-catenin 2* (*ctnnb2)*, *bmp7a*, *alk8*, *smad2* and *smad5*). In addition, ASOs substantially knocked down a lncRNA, *MALAT1*. These results establish ASOs as useful knockdown reagents in zebrafish.

## Results

**Fig 2:**
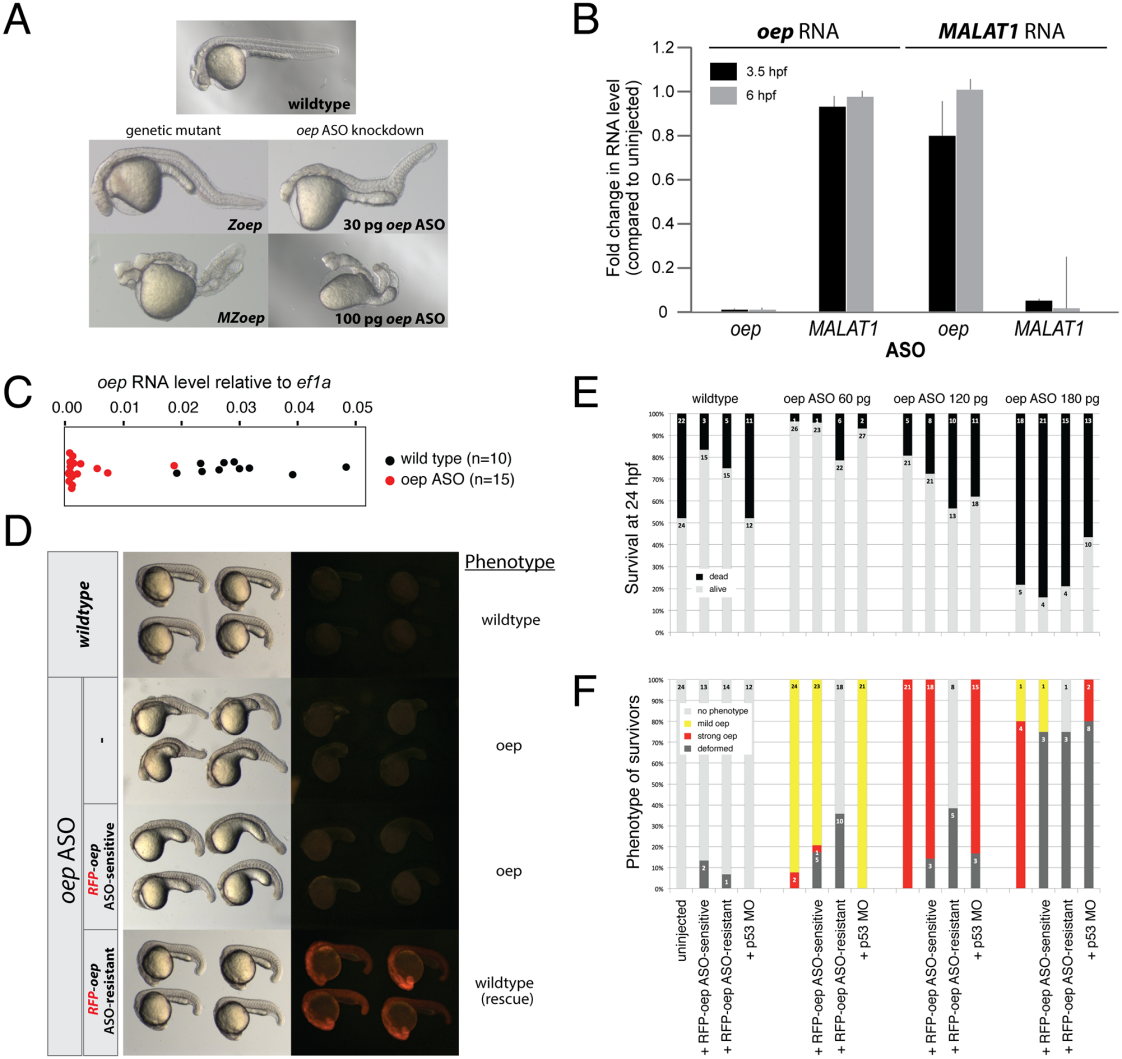
Efficiency and specificity of *oep* ASO-induced *oep* (*one-eyed-pinhead*) mutant phenotypes. A) Injection of *oep* ASO induces dosage-dependent *oep* phenotypes that resemble zygotic (*Zoep*) and maternal-zygotic (*MZoep*) *oep* genetic mutants. B) *oep* ASO and *MALAT1* ASO knockdowns are specific. The RNA levels of *oep* and *MALAT1* were measured by qPCR in *oep* ASO (100 pg) and *MALAT1* ASO (80 pg)-injected embryos. Shown is the fold change in RNA level compared to WT (wildtype), normalized to *ef1a* (error bars: standard deviation of the mean of 3 independent experiments). C) qPCR-based measurement of *oep* RNA levels in individual *oep* ASO (100 pg)-injected (red) or uninjected (black) embryos at shield stage (6 hpf). D) Rescue of *oep* ASO-induced *oep* phenotypes by coinjection of an *oep* ASO-resistant *RFP-oep* fusion mRNA. Note that the *oep* ASO-sensitive *RFP-oep* fusion mRNA is efficiently knocked down (no red fluorescence) and does not rescue. E) Quantitation of survival at 24 hpf and F) quantitation of phenotypic strength in survivors at 24 hpf in the presence versus absence of p53 (*p53* MO-injected embryos) or *RFP-oep* fusion mRNA rescue construct. The number of embryos in each category is indicated.

In order to test ASO efficacy in zebrafish we designed ASOs against the Nodal co-receptor *oep*. *oep* was chosen as a test candidate because it is expressed both maternally and zygotically and has dosage-dependent phenotypes. The complete phenotype only becomes apparent when both maternal and zygotic *oep* (*MZoep*) are inactivated [28]. 5 ASOs were designed against different regions of the *oep* mRNA using *in silico* RNA-folding predictions (see Materials and Methods and S1 Text) [29]. Each ASO was injected at multiple concentrations (1 to 500 pg/embryo) into single-cell zygotes. Two ASOs caused *oep*-specific phenotypes when injected between 30 and 150 pg (Fig 2A, 2D and 2F, S1A and S2A Figs). We found that all ASOs, regardless of their nucleotide sequence, were toxic to embryos when injected above 200 pg, causing deformation and death (S1B Fig). This toxicity was not substantially ameliorated by co-injecting a p53 MO (Fig 2E) [30]. The ASO targeting a 20 nt region close to the 3’end of the *oep* ORF was most effective: injection of 30–60 pg of this ASO resulted in partial loss-of-function phenotypes, resembling partial *oep* loss-of-function mutants, and injection of 100–150 pg of the ASO caused phenotypes indistinguishable from complete loss-of-function *MZoep* mutants (Fig 2A and S1A Fig; quantitation of phenotypes in Fig 2F and S2A Fig (“*oep* ASO 2”)). Quantitative real-time PCR (qPCR) confirmed the efficient and concentration-dependent knockdown of *oep* mRNA: 1–3% of *oep* mRNA remained at 3.5 hours post fertilization (hpf) and shield stage (6 hpf) (Fig 2B). Because a small number of *oep* ASO-injected embryos did not show a specific phenotype at 24 hpf, we tested knockdown efficiencies in individual embryos to correlate variability in phenotype with variability in knockdown levels. We found that the level of *oep* mRNA knockdown across individual embryos at shield stage was in line with the variability in phenotypes at 24 hpf (7/21 strong *oep* phenotype, 11/21 dead, 2/21 partial *oep* phenotype, 1/21 deformed, versus 13/15 ASO-injected embryos with a >3-fold reduction in *oep* mRNA levels) (Fig 2C). The observed phenotype was specific to the knockdown of *oep* mRNA as injection of an *oep* mRNA containing 7 nucleotide changes within the ASO recognition site was able to rescue the ASO-induced phenotype (Fig 2D and 2F). Moreover, quantitation of the levels of *oep* and *MALAT1* RNA in *oep* ASO-injected and *MALAT1* ASO-injected embryos (see below) revealed that each ASO was specifically knocking down the target RNA and not the unrelated RNA (Fig 2B).

**Fig 3:**
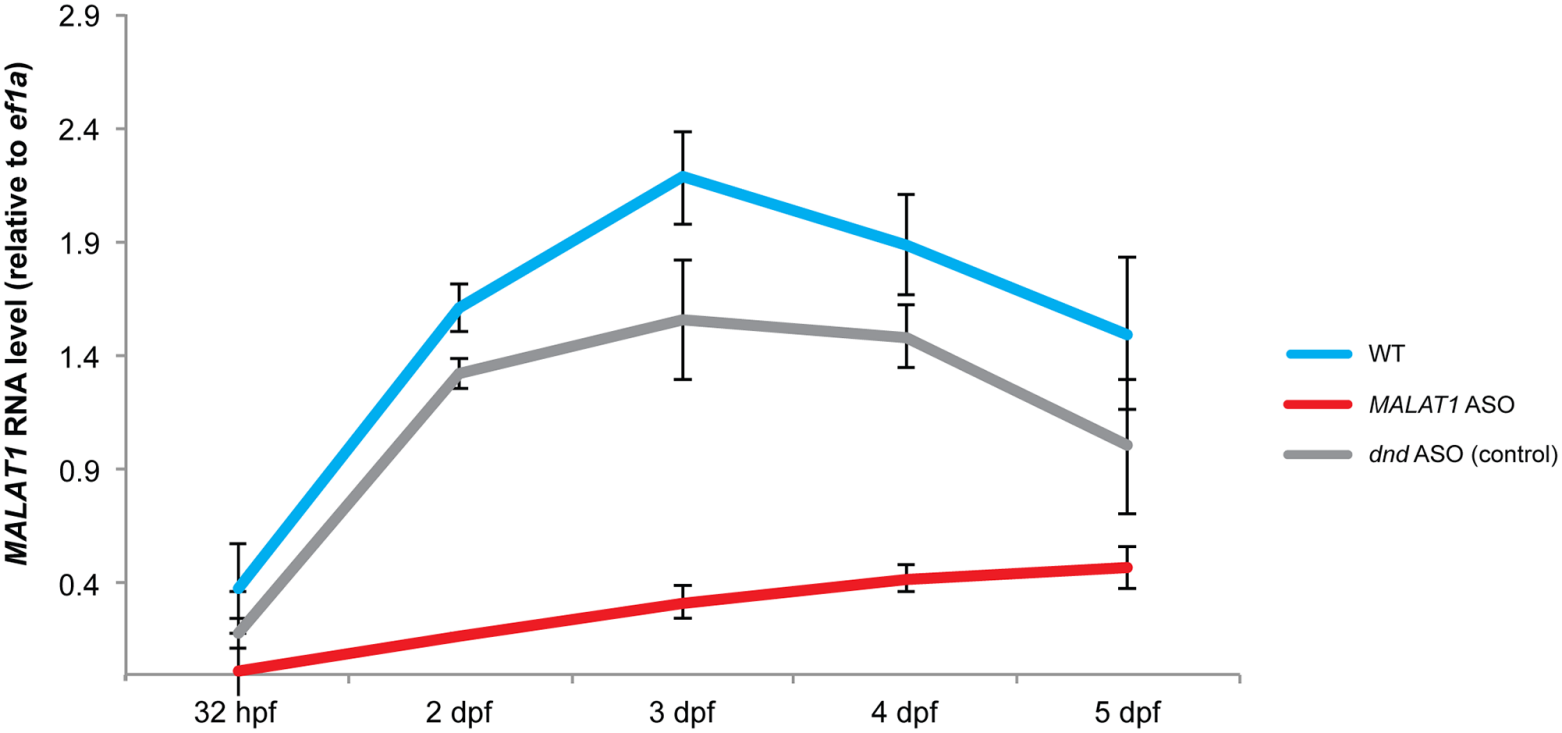
ASO-mediated RNA knockdown persists for several days. *MALAT1* and *ef1a* RNA levels were measured for 5 days post injection by qPCR in uninjected (= wildtype), *MALAT1* ASO (100 pg) and *dnd* ASO (25 pg)-injected embryos. Shown are *MALAT1* RNA levels normalized to *ef1a*. Error bars show standard deviation of the mean of 2 independent experiments (10 embryos each).

To assess the perdurance of ASO-mediated transcript knockdown in zebrafish and to test whether ASOs could be used to knock down non-coding RNAs in zebrafish, we chose to target *MALAT1*. This lncRNA is one of the most highly expressed transcripts during development, it localizes to the nucleus, and has been efficiently targeted with ASOs in mouse models [22]. Notably, 2/3 ASOs against *MALAT1* reduced *MALAT1* transcript levels to 1–10% of wild-type levels (Fig 2B and data not shown), and substantial knockdown persisted for at least 5 days after injection of *MALAT1* ASO but not after injection of an unrelated ASO (*dnd* ASO) (Fig 3). Consistent with the lack of detectable phenotypes in *MALAT1* knockout mice [31–33], development proceeded normally in zebrafish embryos depleted of *MALAT1* RNA (S2A Fig). Nevertheless, the perdurance of *MALAT1* RNA knockdown shows that ASOs can be useful reagents to cause sustained knockdown of zebrafish mRNAs and lncRNAs for several days post injection.

**Fig 4:**
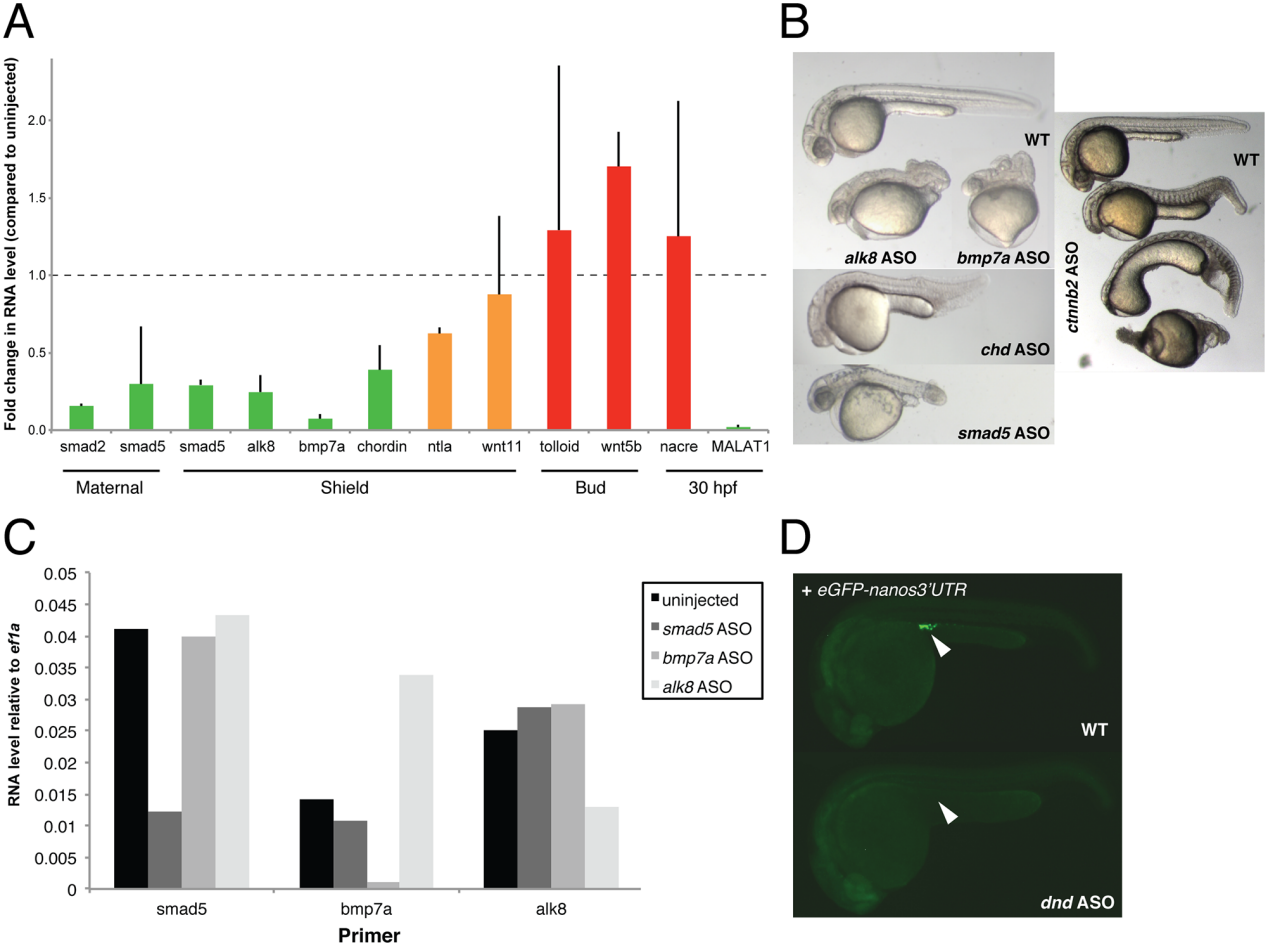
ASO-mediated RNA knockdown correlates with phenotype. A) The RNA level of the gene corresponding to each ASO was measured (compared to WT, normalized to *ef1a*) and correlated to the presence of a phenotype: green = reproduced published phenotype (in case of *MALAT1*: no mutant phenotype), orange = reproduced published phenotype in a smaller percentage of embryos, red = did not produce a phenotype. Injected amount of ASO per embryo: 50 pg *smad2* ASO, 50 pg *smad5* ASO, 50 pg *alk8* ASO, 50 pg *bmp7a* ASO, 150 pg *chordin* ASO, 100 pg *ntla* ASO, 50 pg *wnt11* ASO, 150 pg *tolloid* ASO, 100 pg *wnt5b* ASO, 100 pg *nacre* ASO, 150 pg *MALAT1* ASO. Error bars show standard deviation of the mean of 2 independent experiments (10 embryos each). B) ASO-generated phenotypes for *alk8*, *bmp7a*, *chordin*, *smad5* and *ctnnb2* (shown are 3 embryos representative of the different severities of *ctnnb2* ASO-induced phenotypes). C) ASOs only target the cognate RNA, and not unrelated RNAs. qPCR-based assessment of ASO specificity to their cognate target genes (10 embryos each). D) *dnd* ASOs block germ cell formation. Germ cells were labeled by injection of 80 pg *eGFP-nanos3’UTR* mRNA. Coinjection of 25 pg *dnd* ASO caused complete loss of green germ cells (white arrow).

To test whether ASOs could be used as a general knockdown reagent in zebrafish, we selected an additional 17 embryonically expressed genes with known mutant phenotypes (see Table 1). For 10 of the targeted genes, we assessed whether ASO injection leads to degradation of the target RNA by qPCR. Each ASO was injected at 4 concentrations, and samples were collected at the peak times of the corresponding gene’s expression during the first 36 hours of development to assess target mRNA levels. After monitoring development of the remaining embryos for 24 hours to determine the highest concentration of ASO injection that produced minimal embryonic death, we processed the corresponding samples for qPCR to determine if there was knockdown of the target mRNA. We observed a strong knockdown for *alk8*, *smad5*, *smad2*, *chordin* and *bmp7a*, a partial knockdown for *ntla* and *wnt11* and no knockdown for *nacre*, *tolloid and wnt5b* (Fig 4A). Notably, knockdown efficiency correlated with phenotype. First, ASOs that caused efficient knockdown of their target mRNAs (e.g. *alk8*, *smad5*, *smad2*, *chordin and bmp7a* ASOs) reproduced published mutant and knockdown phenotypes in surviving embryos [34–40] (Fig 4B and S2B Fig; quantitation of phenotypes in S2A Fig). Second, 2 of the 3 ASOs that failed to knock down their cognate mRNAs (*nacre and tolloid* ASOs) did not produce a specific phenotype: they either caused no phenotype or resulted in embryonic deformation and death (S1B Fig; for quantitation of survival and phenotypes see S2A Fig). Results for *wnt5b* ASO, the third ASO that failed to knock down its target gene based on qPCR, were inconclusive due to high variability and high toxicity (data not shown). Third, *ntla* and *wnt11* ASO injection lead to partial knockdown and reproduced the published mutant phenotype [41] in a smaller proportion of embryos (S2A and S2B Fig). Although we found that in most cases a successful knockdown (measured by qPCR) predicted a loss-of-function phenotype, there was a single case in which ASO knockdown achieved up to 90% reduction in the target mRNA, and yet the injected embryos had few gene-specific phenotypes (*oep* ASO#1, S2A Fig). To ensure the knockdown was specific to the ASO that was injected, we assessed the level of *smad5*, *bmp7a* and *alk8* RNA in uninjected embryos and those injected with either a *smad5*, *bmp7a* or *alk8* ASO. Indeed, only the RNA corresponding to the injected ASO was reduced (Fig 4C). Together, these results suggest that qPCR can be a useful assay to pre-screen ASOs for their ability to knock down target mRNAs.

**Table 1:** Overview of ASO experiments.

Gene name (Mutant name)	Expression pattern	No. of effective ASOs/designed	Assay of ASO knockdown efficiency	Reference for published morphology
**PROTEIN-CODING**				
*alk8 (lost-a-fin)*	maternal + zygotic	**2/2**	morphology; RT-PCR	[38,39]
*bmp2b (swirl)*	zygotic	0/2	morphology; RT-PCR	[50–53]
*bmp7a (snailhouse)*	zygotic	**2/2**	morphology; RT-PCR	[34,35,53]
*chordin (dino)*	zygotic	**2/2**	morphology; RT-PCR	[6,36,54]
*ctnnb2 (ichabod)*	maternal	**1/2**	morphology (ventralization)	[55]
*cx41*.*8 (leopard)*	expressed in late larvae + adults	0/3	morphology (spotted pigment pattern)	[56]
*dnd/dead-end*	germ-cell specific	**1/4; 1** * **/4**	morphology (ablation of germ cells at 24 hpf)	[42,43]
*hcrt/hypocretin*	expressed from 1dpf	0/3	in situ (hcrt expressing cells)	[57]
*mitfa (nacre)*	expressed from 1dpf	0/2	morphology; RT-PCR	[6,58]
*oep/one-eyed pinhead*	maternal + zygotic	**2/5**	morphology; RT-PCR	[28,59,60]
*slc24a5 (golden)*	expressed from 1dpf	0/3	morphology (loss of pigment)	[61]
*smad2*	maternal + zygotic	**2/3**	morphology; RT-PCR	[62]
*smad5 (somitabun)*	maternal + zygotic	**2/2**	morphology; RT-PCR	[40,53]
*ta-T/ntla/no-tail a*	zygotic	**2** * **/2**	morphology; RT-PCR	[6,41]
*toddler/apela/ELABELA*	zygotic	0/2	morphology (gastrulation + heart defect)	[63,64]
*tll1/tolloid/mini fin*	zygotic	0/2	morphology; RT-PCR	[53,65]
*wnt11 (silberblick)*	zygotic	**2** * **/2**	morphology; RT-PCR	[53,66]
*wnt5b (pipetail)*	zygotic	0/2	morphology; RT-PCR	[53,67]
**NON-CODING**				
*MALAT1*	highly expressed from 1 dpf	**2/3**	RT-PCR	
*miR-126*	expressed from 1 dpf; endothelia	0/2	morphology (blood formation)	[68,69]

*partial knockdown effect

For list of all ASO sequences, including information regarding effectiveness, see S1 Text.

To test whether ASOs could be an efficient knockdown reagent for transcripts required in only a small subset of cells, we chose to target *dnd*, a germ plasm component required for germ cell migration and survival [42]. Loss of *dnd* functionality leads to loss of germ cells—a phenotype that can be monitored by labeling germ cells with eGFP [43]. Injection of 25 pg of *dnd* ASO resulted in complete germ cell loss at 24 hpf (Fig 4D and S2A Fig), reproducing the phenotype seen with MO-mediated translational inhibition [42,43].

In summary, after targeting 20 genes with 50 individual ASOs (see Table 1), our results reveal that ASOs can be an effective knockdown reagent for protein-coding and noncoding transcripts.

## Discussion

This study reveals that ASOs can be an effective RNA knockdown reagent for zebrafish. Three observations establish the suitability of ASO use in zebrafish. First, ASOs are specific, because (i) we can rescue the phenotype caused by ASO-mediated knockdown of *oep* with an ASO-resistant *oep* mRNA, and (ii) ASOs cause substantial reduction of the target mRNA without corresponding reductions in unrelated mRNAs. Second, ASOs can target maternal, zygotic, nuclear and cytoplasmic coding and noncoding RNAs, since we were able to target maternal *oep*, *smad5* and *dnd*, zygotic *oep*, *chordin*, *bmp7a and alk8*, and the nuclear noncoding RNA *MALAT1* [44]. Third, unlike MOs, ASO-mediated knockdown can be quantified by qPCR, which generally allows phenotypes to be correlated with a reduction in expression of the target RNA.

Although our experiments highlight the power of ASOs to efficiently knock down target RNAs, there are three limitations associated with ASO use. First, ASOs are toxic to the embryo when injected above 200 pg, irrespective of the nucleotide sequence. The toxicity is usually manifested as cell death followed by embryonic lethality. Thus, high levels of ASOs should be used with caution if the resulting phenotype could be linked to general toxicity in the embryo [45]. Second, ASOs appear to only be effective in a narrow concentration range. We found that some ASOs produced no knockdown at moderate levels (e.g. 100 pg) but were toxic and induced lethality at higher levels (200 pg). Therefore, it is important to inject at least 2–3 different concentrations to capture the ideal concentration range and produce efficient target knockdown. Third, not all ASO designs produced knockdown phenotypes. We designed 2–5 ASOs for each gene but, with a few exceptions such as *oep* and *dnd*, we generally found that either all or no ASOs were effective for a particular gene. Of the 50 ASOs we designed for 20 genes, we found that 21 (42%) effectively targeted 11 genes, of which 16 (32%) caused loss-of-function-like phenotypes and 5 (10%) caused partial knockdown phenotypes (see Table 1). Steric-blocking MOs are only effective when targeting sequences around the translational start, limiting design options. By contrast, ASOs designed against any region of the target RNA can lead to target degradation. One current limitation of ASO design is the lack of an effective target prediction algorithm. Currently, ASOs are designed using antisense reagent target prediction strategies [46] that have limited predictive power (MAB, personal communication), necessitating the need to empirically test each ASO for knockdown efficiency. However, as ASO use becomes more widespread, target prediction tools will likely become available. A good example of such a development comes from the RNAi field, where machine learning algorithms have been trained to improve siRNA knockdown rates [47].

Due to these limitations, ASOs—like all knockdown reagents—should be used with caution and with appropriate controls. ASOs allow fast, cost-effective and preliminary assessment of gene function, but we do not recommend that ASOs be used to firmly establish the function of previously uncharacterized genes, unless phenotypes are confirmed with genetic mutants [48,49]. Where mutant phenotypes are known, ASOs and other knockdown reagents that recapitulate the mutant phenotypes can be valuable, for instance to knock down maternal transcripts or non-coding RNAs, or create clutches of embryos that all have the same or similar phenotypes. Thus, ASOs are a useful addition to the zebrafish knockdown reagent toolkit.

## Materials and Methods

### Ethics statement

All vertebrate animal work was performed at the facilities of Harvard University, Faculty of Arts & Sciences (HU/FAS). The HU/FAS animal care and use program maintains full AAALAC accreditation, is assured with OLAW (A3593-01), and is currently registered with the USDA. This study was approved by the Harvard University/Faculty of Arts & Sciences Standing Committee on the Use of Animals in Research & Teaching under Protocol No. 25–08.

### ASO design

ASOs were manually designed to target regions of the RNA predicted by *in silico* methods [29] to have no substantial secondary structure. For ASO DNA domains (central 10 bases of an ASO) with CpG, 5’Me-dC was used instead of standard deoxy-Cytosines (dCs) to protect the ASO from potential methylation by TLR9. 2–5 ASOs were designed for each target gene. For genes with published, successful MO-induced phenotypes, one ASO was designed to target a region overlapping the MO binding site, with the exception of *oep*, *MALAT* and *mir-126*. For the majority of genes, further target regions were chosen within the 5’UTR or very close to the ATG translational start codon. A complete list of cDNA sequences of target genes with annotated MO and ASO sites can be found in the Supporting Information (S1 Text).

### Microinjection, RNA purification and qPCR

Zebrafish TLAB strain zygotes were collected and injected through the chorion with 25–200 pg of an ASO. ASO injections above 200 pg resulted in general toxicity. Co-injection experiments with *p53* MO included 2.6 ng of *tp53* MO (5’-GCGCCATTGCTTTGCAAGAATTG-3’) [30]. Each batch of ASO-injected embryos was assessed individually for knockdown/loss-of-function phenotypes of the cognate gene by scoring embryonic morphology, performing qPCR, reporter gene expression (GFP-nanos-3’UTR for *dnd* ASO) or *in situ* hybridization of marker genes (for *toddler* ASO and *hypocretin* ASO; data not shown). For morphological assessment, embryos were raised to 24–30 hpf and imaged. For qPCR-based assessment of knockdown efficiencies, total RNA was isolated from 5–10 embryos of the appropriate developmental stage using the standard TRIzol (Invitrogen) protocol. Genomic DNA was removed by TURBO-DNase treatment. For reverse transcription (iScript, BioRad), equal amounts of total RNA per sample were used as input (100–500 pg of total RNA, depending on the experiment). 1 μl of a 20 μl cDNA reaction (equivalent to 0.05–0.25 pg of total RNA) was used as template for quantitative real-time PCR (qPCR). qPCR reactions were run on a Stratagene MX3000p using GoTaq (Promega) and 0.25 μM of gene-specific forward and reverse primers (see primer list below). qPCR cycling conditions: 10 min 95 at degrees Celsius, followed by 45 cycles of 30 sec at 95 degrees Celsius, 30 sec at 55 degrees Celsius, and 20 sec at 72 degrees Celsius. qPCR reactions were performed in triplicate and averaged. For each gene, gene expression levels were calculated relative to a reference gene, *ef1a*. Knockdown efficiencies were calculated as the ratio of normalized gene expression in ASO-injected versus uninjected (or non-cognate ASO-injected) sample. Each experiment was performed at least in duplicate, using independent biological samples.

### *oep* ASO rescue

To generate an *oep* ASO-resistant *oep* mRNA rescue construct, 7 nucleotides within the targeting site of the most efficient *oep*-targeting ASO (designated ASO#2 in S1 Text; ASO#2 sequence: mG*mG*mC*mG*mA*A*C*A*T*G*A*C*A*A*T*mU*mG*mU*mA*mG (* denotes phosphorothioate bonds; ‘m’ denotes 2’O-Methyl RNA nucleotides)) were mutated by standard PCR-based site-directed mutagenesis. In brief, overlapping fragments encoding the 5’ portion (Forward primer: SP6 ATTTAGGTGACACTATAGA; Reverse primer: cACgATgGTgctcgacagtgttctgagggagcccgaccgg (capital letters denote nucleotide changes) and the 3’ portion (Forward primer: aacactgtcGAGCacCatCgtGatgttcgctgcttttattttacaccg; Reverse primer: T3 AATTAACCCTCACTAAAGG) of a fusion between *oep* and *RFP* (*oep signal peptide–RFP-oepORF*) were amplified and fused together by PCR, using standard methods. mRNAs of ASO-sensitive and ASO-resistant *oep-RFP* fusion constructs were synthesized using SP6 mMessage Machine (Ambion) and injected either with or without 100 pg of *oep* ASO#2. Rescue ability was assessed by 1) strength of *oep* mutant phenotype; and 2) persistence of red fluorescence in the presence of *oep* ASO#2.

### Imaging

Fluorescently labeled embryos (mRNA injection of *eGFP-nanos3’UTR* [43] or *RFP*-*oep* (*oep* ASO-sensitive or -resistant constructs)) were imaged on the Zeiss Discovery Scope V12, and brightfield images were captured using the Leica MZ16F.

### qPCR primers

*ef1a* was used as reference gene (ef1a_F agaaggaagccgctgagatgg, ef1a_R tccgttcttggagataccagcc). The following primers were used to amplify specific target genes: alk8_F cgttatcattagcaatgatgtgacg, alk8_R tcttcttttcttctggacttgtgag; bmp2b_F agttttcatcacgaagaggctt, bmp2b_R ttaattctgtggaagccactcg; bmp7a_F agctttgcgaatacagtggatc, bmp7a_R ctgacatggaaggtctcgttttc; chordin_F gttcctctggccggtgttctggt, chordin_R ctcctctggggttcatcttggtgct; MALAT-1_F aaggggatctgcacttttctctctcttct, MALAT-1_R cacacaaacacttccaccacacacc; nacre_RT_F ctcaactgtgagaaagagatggac, nacre_RT_R gttactgatggaaactccagctg; ntl_F aatctggatattcacaactcggtg, ntl_R agttgtccatgtagttattggtgg; oep_F gaatgacgagtcaactgttcgggttc, oep_R tcttgcagcaggtacggctttgtt; smad2_F Aagcggagcaggaggtggtggag, smad2_R gtccccaaatttcagagcaattgctgg; smad5_F gtagggtgagtttggagagatg, smad5_R gtagggtgagtttggagagatg; tolloid_F aaatggtccccaggcaatatc, tolloid_R agttatactcctgacctggctg; wnt11_F gacctcaagtctaaatacctgtcg, wnt11_R gtcttgttacactgcctgtctg; wnt5b_F cgtcatgcatataggcagcc, wnt5b_R cgaagcggtagccatagttg.

## Supporting Information

S1 Fig Specificity and toxicity of ASO-mediated RNA knockdown in zebrafish embryos. A) *oep* ASO-mediated *oep* mRNA knockdown induces *oep*-specific phenotypes with high efficiency, while MALAT1 ASO-mediated *MALAT1* lncRNA knockdown does not induce any visible, gene-specific phenotypes (3 dead (= black) embryos). B) ASOs induce toxicity at higher concentrations. The concentration at which a specific ASO is toxic for an embryo varies and is ASO sequence-dependent. In general, injection of > 200 pg of an ASO results in general toxicity. Shown are representative images of ASO-injected healthy (= no phenotype), deformed and dead embryos during mid-gastrulation (70% epiboly) and at 24 hpf.
(TIF)

S2 Fig ASO-mediated RNA knockdown is applicable to multiple different transcripts. A) Summary quantitation of survival and phenotypes of ASO-injected embryos at 24 hpf. Percentage plots are derived from multiple independent experiments (at least 2 independent experiments per ASO). Compiled numbers of embryos scored for each ASO are indicated. B) Representative images of *smad2* ASO (left) and *ntla* ASO (right)-injected embryos at 30 hpf. *ntla* ASO caused a gene-specific phenotype only in a subset of embryos (left: overview image with phenotypic and non-phenotypic embryos; right: higher magnification view of phenotypic embryos).
(TIF)

S1 Text Annotated target sequences. Shown are the cDNA sequences of target genes (translational start codon (ATG) in capital letters) and ASO (turquoise and green (most efficient) highlights) and MO (blue) targeting sites.
(DOCX)
