# Supplementary figures and images for: Antisense Oligonucleotide-Mediated Transcript Knockdown in Zebrafish
Source: PLoS One. 2015 Oct 5;10(10):e0139504. doi: 10.1371/journal.pone.0139504 (PMC4593562; doi:10.1371/journal.pone.0139504)

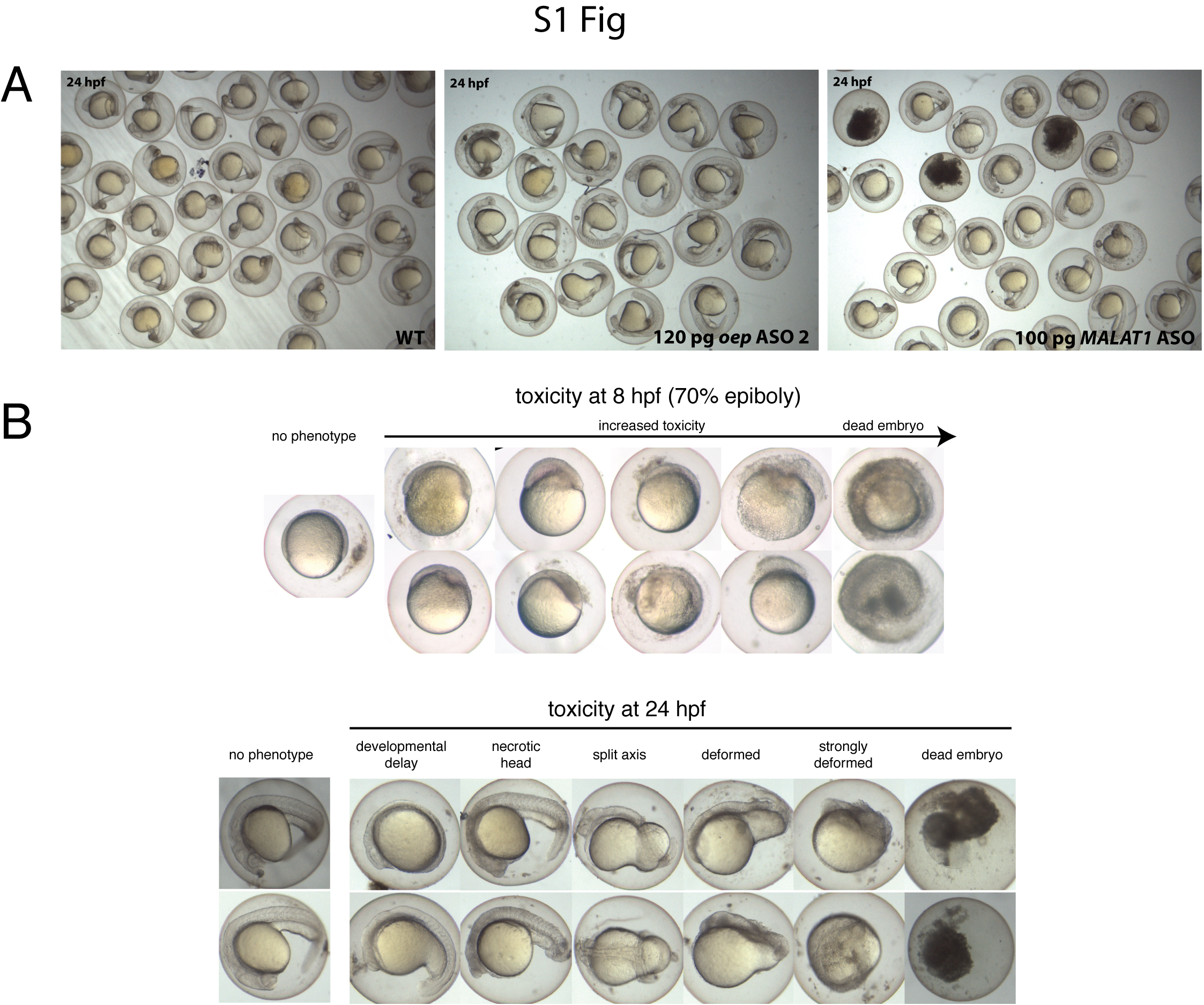

Supplement: S1 Fig — A) oep ASO-mediated oep mRNA knockdown induces oep-specific phenotypes with high efficiency, while MALAT1 ASO-mediated MALAT1 lncRNA knockdown does not induce any visible, gene-specific phenotypes (3 dead (= black) embryos). B) ASOs induce toxicity at higher concentrations. The concentration at which a specific ASO is toxic for an embryo varies and is ASO sequence-dependent. In general, injection of > 200 pg of an ASO results in general toxicity. Shown are representative images of ASO-injected healthy (= no phenotype), deformed and dead embryos during mid-gastrulation (70% epiboly) and at 24 hpf. (TIF) [file pone.0139504.s001.tif]

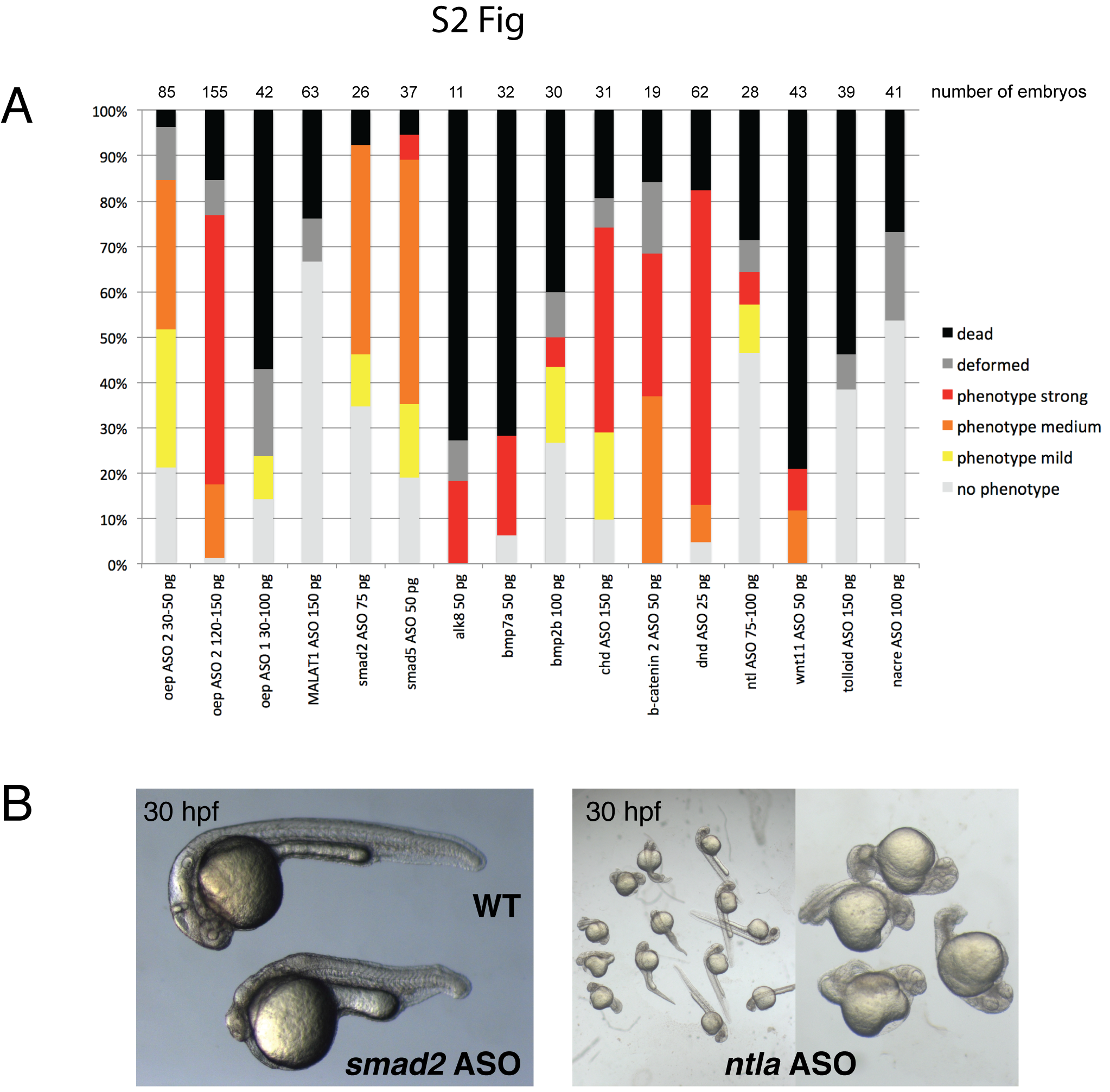

Supplement: S2 Fig — A) Summary quantitation of survival and phenotypes of ASO-injected embryos at 24 hpf. Percentage plots are derived from multiple independent experiments (at least 2 independent experiments per ASO). Compiled numbers of embryos scored for each ASO are indicated. B) Representative images of smad2 ASO (left) and ntla ASO (right)-injected embryos at 30 hpf. ntla ASO caused a gene-specific phenotype only in a subset of embryos (left: overview image with phenotypic and non-phenotypic embryos; right: higher magnification view of phenotypic embryos). (TIF) [file pone.0139504.s002.tif]
