# Supplementary material for: Antisense Oligonucleotide-Mediated Transcript Knockdown in Zebrafish
Source: PLoS One. 2015 Oct 5;10(10):e0139504. doi: 10.1371/journal.pone.0139504 (PMC4593562; doi:10.1371/journal.pone.0139504)
Supplement: S1 Text — Shown are the cDNA sequences of target genes (translational start codon (ATG) in capital letters) and ASO (turquoise and green (most efficient) highlights) and MO (blue) targeting sites. (DOCX) [file pone.0139504.s003.docx]

**S1 Text**

Annotated target sequences.

**Key**

mA, mU, mC, mG 2’O-Methyl (2’OMe)-modified RNA nucleotides

iMe-dC methylated deoxy-Cytosine

* phosphorothioate bond

**ATG** translational start

MO targeting site blue text

ASO targeting site turquoise or green (best ASO knockdown efficiency) highlight

***alk8/acvrl1***

MO GATTCATGTTTGTGTTCAATTTCCG

Alk8 2’OMe ASO#1 (GC = 35%)

mC*mA*mU*mG*mU*T*T*G*T*G*T*T*C*A*A*mU*mU*mU*mC*mC

Alk8 2’OMe ASO#2 (GC = 55%)

mG*mU*mG*mC*mU*G*C*A*A*T*G*C*C*C*C*mA*mU*mU*mU*mG

>NM_131345.1

gtccggcagacagtctgtcagcgctgaaaacacgacttcgaataaaagagtgatattacggaaattgaacacaaacatgaatctgcattgcagctaaaggatccaatgggaagacagagtctggctgtgcgaatactgaaaggcagcgctgagagtcacttgaggagttgtacaatgcattgaaccaa**ATG**gggcattgcagcacccaaatcatcatcctgttcctgcttcagttcttacagacatcagctaaagatgtctccattgactgcatgtgtgtcggcagtgactgtaatgagcagcagtgtactggtgaccagtgttacacctccgttatcattagcaatgatgtgacgacgttcaagcggggctgcttgatcgggccggcgagcaagcgcatgacctgctccgcaacagcttctgctagtcatgtggtagaatgctgttctcaacacatgtgcaacgccaacgtctccaaagagaccctacttcgactgctgctcacaagtccagaagaaaagaagactgttcattaccgcgtggaaatgttggttctgtttgtgttggggccgtttgtggttctgggtctgctgtcttttctggctttgctggtgtgccgtcgactccatcatgggcgtctggagagactgcacgagtttgacactgaacagggggccatcgatgggcttatcgcgtctaatgttggagacagcacacttgcggatctgatggatcactcctgcacttcaggcagtggttcaggactgcccttcctggttcagagaacggttgcgcggcagatcagcctggtggagtgtgttggtaaaggacggtacggtgaagtgtggagaggtcaatggcaaggagaaaatgtagccgtgaagatcttttcctctagagatgagaagtcatggtttcgagaaacagaaatttacaacactgttctgctacgacatgaaaatatattaggcttcatggcttctgacatgacctcccgaaactctagcactcagctgtggctgatcacacactatcacgagaatggctctctgtatgactacctgcagcgtgtggctgtggagatggcagatggactgcacatggcggcgtcgattgccagcggactggtgcacctgcacacggagatctttggcacggagggcaaaccggccatcgctcacagagacctgaagagcaagaacatccttgtgaagaaagatttgcagtgctgcatcgctgacctgggtctggcagtaacacacacgcagtctgataatcagcttgatgtgggaaataatcctaaagtgggaaccaaacgctacatggcaccggaggttctagatgagaccattcagacggactgttttgacgcctataagagggtggatatctgggcctttgggttggtgctgtgggagatcgcacgcagaaccatcagcaatggaattgtagaggaatacaagccgcctttctatgacctggttcctaatgatcccagctttgacgacatgaggaaagtggtttgtgtggagcagcaaaggccattcattcccaaccgctggttttcagatcctaccctgtctgctctggtgaagctgatgaaagagtgctggtaccagaacccctcggctcgtctcactgccctgcgcatcaaaaagactctggataaaatccacagttcactggagaagggcaaaaccgactgctgaggagagagagagctgcagaactactgaccgctcacccgctcattatacagacactgtgtgtaaccaaaccattggattccagcgctggcatctgtcccaactctaacatgctgtttatttgctttattcttcttgttcattggcctaaaaacttttttatagagaattacgctctcttttatggattttatttttccccccactgaacgcggtgtaactcacatttaccctcaagaagcaacaaaaaggacagagacgccatcaaatatgtggacgcacactgacaggccttgaagaaaccctctgtcatggatttttttaacagcatcatgttgtctatttaaccattggcagactttaaagatttgtacttttcagaaaaatgaaaaggacggatacacacatgctgaaatgcaatcagtctcataatgtgtgtgtgtgcacatgattcttgttacaaccagaaaaacttcatagctgtaacatcacctgcttttagacatctggtgggttttgagctttttaaaggtcctgatagttcttgtagataggttaggcaaagttagtcacagttttgctatatggacttatcataaaaccggttggttgcttaatgccactatgtacagtaggaatcaacaggcttctttctttaattaggcctgtttagtgaccagacctgggttttgaactagtggtaaaaaaaaaaaaaattctgtcatcatgtaattccaaaaataaataactttttcagtgggaaaaacacagaaaaagatgttttaaagaatgctcacgctgctctttttccatacagtgagcacatattgtggcatgctctctaaaaattaacgaaaaccgttcatatattgtttggaatgacatgaatgatgagagaataattttttttatatatatatatatatatttttgtttgggtgaactcgcagtctggtcattctcatagcaccctatgagggcttccctttgacacaaggcctttctgacctgttgcctctatgcctgcctgactaaaagccattgtagcacaccgtagtgcttttttgttttatttttttgtagctgttgtctttatttttgccctcacaggcatgcgtacttgatgtttgttcaatgacaatcagccgctgtccagtagggcctgaactatttgctgtcatgtacaaaaccctacttatgagcttttaaggtgacgtctcagcacactaacagagctagtcatgatcataaatagactctggttttgtgaatttgcgtatttgtttaccctcatgcttttttcacactttttgtatcacagttttcaacacgcaccatgagccgagagaattcggatgcttttaaaactttaaacgctttttgactatttaaaagtgacactctaatataggatggaagaggtttacaaaggaaaatgctattgttgtataccgaatggcttgtgtatgttgctaatggtttgggtaaagtgaaatggtttgaatttaatttttttaatttgggaaaaatgggtcctgatacactacaactttcacttctggtcgcttttgctctcctataaaaccaaacgtttacatgtttgtgttgacctggggagcgatgttaggaattaagactacagcagctattgcattatgaactgtgaatgttataatgaagaaaccttatgtatgatgttactggaatactgaatgcgggttaagaattaaaaggaaatctgctttttttcaaaaaaaaaaaaaaaaaa

***bmp2b***

MO CGCGGACCACGGCGACCATGATC

Bmp2b 2’OMe ASO#1 (GC = 65%)

mG*mG*mA*mC*mC*A*/iMe-dC/*G*G*/iMe-dC/*G*A*C*C*A*mU*mG*mA*mU*mC

Bmp2b 2’OMe ASO#2 (GC = 60%)

mA*mC*mA*mG*mA*G*A*G*T*/iMe-dC/*G*T*/iMe-dC/*G*C*mA*mG*mC*mU*mC

>NM_131360.1 gaattcggcacgaggcgtgctgcacgcagagatgagtctccaaacagccacggaaaacttctgctgaccacaagttttcgatttctttaaaacaaaaacaaaaaatgacaaatccaggattgtgcgatctcgcgctgtcacttttgggattgctgctgtctttgacctgagcgctcgcgcacttcattagagtttagtagagtctagtctgaagtgttgcacaagtatgaacaagaagaggcgacttgagctgcgacgactctctgtcgtgggataaaaaatcgcttgtggattaaacacgaattcatgaggaacttaggagacgacgggaacgcagaccggccacagcgcttcctcctccggaactgactgatc**ATG**gtcgccgtggtccgcgctctcacggtgctgttgctcggtcaggtgttgctgggaggtgccgttggactcattcccgagatcgaccgacggaaatacagtgattcggggagacacacaccggagcgaactgatacaaacttcctgaacgagtttgagctacgcttgctcaatatgttcggattgaagcgaaaacccaccccaagcaaatcggcagtggtccctcagtacatgctggacttgtattatatgcactctgaaaacgatgacccgaacattcggcgcccgaggagcactatgggaaaacatgtagaaagggcagccagcagagcaaacacgatacgaagttttcatcacgaagaggctttcgaggcactgtccagcctgaaaggaaaaacaacgcagcagtttttcttcaaccttacctccattcctggcgaggagctgatctccgctgcggagctgcgcattttcagggaccaagttctcggagatgccagtacgagtggcttccacagaattaacatttacgaggtgttcaggccagctttggccccctccaaagagcctctaaccagacttctggacacccgtctggtgcaggactctcacacgcgctgggaaagcttcgacgtgggttcagctgtggcacgctgggcccgcgaatcccagcacaaccacgggctccttgtagaggtgctccatcctaaggagtcagaagtatccgaggaggctgagagcaaccggaggaagcacgtgagggtcagtcgttcccttcacgcggatgaggactcgtgggcacaagcccgacctctgctggtaacctacagccatgacggtcaaggcacagccgtcttgcattcgaaccgagaaaagcggcaggctcgacgagggcaaaagccgaggagaaagcaccaccagcgctcgaactgtaggcgacatgctctctatgtggacttcagtgatgtcggctggaacgagtggatcgtggcaccgccaggctatcatgctttctactgccatggcgagtgtccgttccctctgccggaccatctaaactccaccaaccatgccattgtccagacgctggtgaactcggtcaactccaacattcccaaagcctgttgcatcccgacggagctcagccctatctcactgctgtacctggacgagtacgagaaggtcattcttaaaaactaccaggacatggtggtggagggctgtgggtgccgatgagaacaatctccccaatgaagacttttatttatacaaaagagcgagctatttggaggaagaaaagaaatatatatgaatatatttatgttgaatgaacaaaacaaaaaaaaaaaaaaaaaaa

***bmp7a***

MO GCACTGGAAACATTTTTAGAGTCAT

Bmp7a 2’OMe ASO#1 (GC = 30%)

mG*mG*mA*mA*mA*C*A*T*T*T*T*T*A*G*A*mG*mU*mC*mA*mU

Bmp7a 2’OMe ASO#2 (GC = 40%)

mG*mC*mA*mU*mA*A*C*A*T*G*A*A*G*T*T*mG*mA*mG*mU*mC

>NM_131321.2

gagaccgtcagttcaacctcagatcatgctggatgttgctctggattggattaatcaacacgtgtatggcatttaatacagactcaacttcatgttatgctcaaacgaaataatancaatacttcactaatcgattgtggacttctgcntaactcttgttattttttttaagaatat**ATG**actctaaaaatgtttccagtgcatcttatttccactttgatagtggcttgtggttgcagtgtgctcgcagacgcaatgcaagcaaacttcaccatggataatgacattcagtctagcttcatccagcggcgcctgaagagccaggagcgcagagagatgcagcgggaaatcctctctatcctcgggctgccgcaacggccacgtccgctcctgcacgagagacacaccgcggctcccatgtacatgctggatctgtataacgccatcctggaggacggagacnggcgcggtgggcttgtgtactcttatgaacccgcgtacacgaccccgggacccccgctggtgacccagcaggacagtcgctttctcagtgatgccgacatggtgatgagctttgcgaatacagtggatccagaagaagaccttcaactgtatcatcagcatcgcagagagttcagatttgacctttctcgcatcccgccaggtgaaactgtgaccgctgcagagttccgaatctacaaagactttgttcgcgagcgttatgaaaacgagaccttccatgtcagcgtgttccaggtgcttcaacagcagcacagaagggagctgtacctgctggactcacgagtggtatgggctgcagaagagggatggctggtctttgacctcacggtcaccagtaaccactgggttataaacccaggtcagaacttaggactgcagctcttagtggagacctcacatggtgcaagaatgaacccaagaagagctggcctggtgggcagcagtggagctcagaataagcagccgtttatggtggcgtttttgaaagcatcaggaatccacctccgcagtgtccgctcggcatcaggaggcaaacagaaaggtcatcaccgcactaaaaacgccaaacctggcgctgcacacagccaggtggctttgaaaacagctgaagccacagagggtgccagcatagatcccaaacagggctgcaaaaagcatgaactctacgtcagcttcagagatctgggatggcaggactggatcatcgccccagagggttatgccgcgtactactgtganggcgaatgtgtgttccctttgaactcttatatgaacgccacaaaccatgctattgtacagacactggttcatttcataaaccctgaaactgtcccgaagccctgctgcgctccaactcagctgcacggaatctcagttttgtacttcgatgacagctccaacgtaatattgaaaaagtaccgcaacatggtcgtcagagcctgcggatgccactaatcactacctctaccctcaccagngggattcaacaagggatttaaagacttgtattcttgattatcaagagcgcagagagaatacgagactcgctgctggacccaaactgganttcttcatatgaagcggataagacaaaactcaaaactcctagaacacagctgagtgtgtgttgtgtctggctttcatttctgcacagagagccaagtttaaaatatcaaaaaggacataagcagatncagagagaaaataaaagagatttaaccagactgatcagggaagatgcagtggatttaagcataatgctatcgtatttcataaaccacttgacttgatttctgaaagaataataccttttaaaaagtctgagaccaaagccgaatt

***chordin***

MO ATCCACAGCAGCCCCTCCATCATCC

chordin 2’OMe ASO#1 (GC = 60%)

mA*mC*mA*mG*mC*A*G*C*C*C*C*T*C*C*A*mU*mC*mA*mU*mC

chordin 2’OMe ASO#2 (GC = 60%)

mU*mC*mC*mG*mG*A*G*C*T*C*T*G*A*C*T*mG*mG*mA*mG*mU

>NM_130973.2

cgcaccgacactacaaaacggttacatacacgcagaaaactccagtcagagctccggaactcgccgaactacactttctgagagaatattgtcgatctgtgcagtattagtagcgatagtgtttgtctgaggagttgaaggtggtgtgg**ATG**atggaggggctgctgtggattctgctgtccgttattatcgcatctgtgcacggatcgagactcaagacacccgcgctgcccatccagcccgagagagaacccatgatttccaaaggattatccggttgctccttcggtggccgcttttactctctggaagacacgtggcatccagatctcggagagccgtttggtgtgatgcactgcgttatgtgtcattgtgagccgcagaggagccggcgagggaaggttttcgggaaagtgagctgcaggaatatgaaacaggactgtccagatccgacctgcgacgatccggtcctgcttcccggacactgctgcaaaacatgcccaaaaggcgactctgggaggaaggaggtcgagtctctgtttgatttcttccaggaaaaagatgacgatttgcacaaatcctacaacgacagatcgtacatcagctccgaggacaccagcactcgagacagcaccaccaccgattttgtggcgctgctgacgggtgtgacagactcttggctgccgagctccagtggtgtcgcgagggccagatttacactttctcgaacaagtctgactttctccatcactttccagagaataaacagaccgagtctcattgcatttctggacactgacggaaacaccgcgtttgagttcagagtcccgcaggccgataacgacatgatctgcggcatttggaagaatgtgccgaaacctcacatgcggcagctggaggcggagcaactgcacgtctccatgaccaccgctgacaacaggaaggaggagcttcagggcagaataatcaaacacagagcactgtttgctgaaacgttcagtgcaatcctgacatctgacgaggtgcattctgggatgggtggaatcgccatgttgacgctcagtgacaccgaaaacaatctgcatttcatcctgattatgcagggacttgttcctccagggagctccaaggtgccagtgcgagtgaaactgcagtatcgacaacacctgctgagagaaatacgagcaaacatcactgcagacgactcagactttgctgaagtgctggcggatctgaacagtcgtgagctcttctggttgtcccgcgggcagctccagatctccgtccagactgaaggtcaaaccctgcgtcacatctcgggattcatttctggaaggagatcatgtgacactctgcagagtgtgttgtcgagcggcgcggctctgactgcgggtcagacgggcggtgtgggctctgctgtcttcactctgcacccgaacggctctctggattaccaactgctggtggcgggtctgagcagcgcggtgctgagcgttagcatcgagatgaagccgcgtcggcgcaacaaacgcagcgttctgtacgagctctcggcagtgttcacagaccagcgggcggcaggcagctgtggacgcgtggaggccagacacacacacatgctgctgcagaacgagctgttcatcaacatcgccaccgcactgcagcccgacggggagctgcgcggacagatacgcctgctgccatacaatggactggacgcgcgcagaaacgagttgccggttcctctggccggtgttctggtgtctccgccggtgcgcactggtgcagcgggtcacgcctgggtctcggtggaccctcagtgtcatctgcattacgagatcatcgttaacggactcagcaagagcgaagacgcctccatcagtgctcacctgcatggattggccgagatcggagagatggacgactcttccaccaatcacaagagactcctgactggtttctacgggcaacaggctcaaggcgtcctgaaggacatcagtgttgaattattgcgtcatttaaatgaaggtacggcgtatctgcaggtcagcaccaagatgaaccccagaggagagattcgcggacggatccacgtcccgaaccactgtgagtccccggcgccccgcgctgagtttctggaggagccggagtttgaggatctgctgttcacacgggagccgacggagctgcgcaaagacacacacactcacgtacactcctgcttctttgagggagaacaacacactcacggctcacagtggacaccgcagtacaacacctgcttcacctgcacctgccagaaaaagacggtgatctgtgacccagtgatgtgtcctacactctcctgcacacacaccgtccagcctgaagaccaatgctgtcccatctgtgaagagaagaaagaatctaaagagacagccgctgtagaaaaagttgaggaaaatcctgaaggctgttatttcgaaggcgatcagaaaatgcacgcacctggaacaacatggcatcctttcgtcccgccgttcggctacatcaagtgtgcagtctgtacctgcaagggctcgacaggggaagtgcactgcgagaaggtgacgtgcccgcccctcacctgcagccgaccaatcagacgcaacccttcagactgctgtaaggagtgtcctcctgaagaaacgccccctctggaggacgaggagatgatgcaggcggacgggacgcggctctgcaaattcgggaaaaactactatcagaacagcgaacactggcatccgagcgtcccgctggtgggggagatgaagtgcatcacctgctggtgtgaccatggcgtcacaaagtgtcagaggaagcagtgtccactgctgagctgcagaaaccccatccgcacagagggaaaatgctgtccggaatgcatagaggacttcatggagaaagaagaaatggcaaagatggcggagaaaaagaaaagctggagacactgacactctctaaagtggagatcaggccgtctcaaacgcacaagcagacgcctgtggactgcacaaacacacacagaagagctccaggacgcgacaaaactacacaacggattattccagaatcaatctcagctgctggaggtgtttggattttcccaccggcgggaatgtgcctttgactgttgtttgtatatttgacttggac

***ctnnb2***

MO CCTTTAGCCTGAGCGACTTCCAAAC

ctnnb2 2’OMe ASO#1 (GC = 60%): most efficient knockdown

mC*mC*mA*mA*mA*C*C*T*G*A*A*C*/iMe-dC/*G*G*mA*mG*mA*mG*mC

ctnnb2 2’OMe ASO#2 (GC = 65%)

mA*mC*mU*mG*mC*C*/iMe-dC/*G*T*C*C*A*G*A*T*mC*mU*mG*mC*mC

>NM_001001889.1

agcgagagcggagagaactgacgcaccgtgaccggagagaataacgagaaacaccggtggattcactgcttacaagacaccaacgagaaggtcaggctccacggccgctgcacggagctctccggttcaggtttggaagtcgctcaggctaaaggattgacgcaacg**ATG**gctagccaggctgacctgatggagctggacatggccatggagcaggaccgtaaggctgcagtgagccactggcagcagcagtcctacctggattcagggattcactcgggcgccaccaccactgcgccctcgctctcaggcaaaggaaacccagaggaagaggatctggacaaccaggtgctgtacgagtgggagcagggcttcagccagcccttcacacctgagcaggtggcagatctggacgggcagtatgccatgacgcgggcgcagagggttcgagccgccatgttcccagagactctggatgagagcgtcccgatggcctccacccagttcgactctgctcatccaacaaacgtacagcgtctggcggagccgtcgcagatgctgaagcatgcggtggtcaacctcatcaactaccaggacgatgcagagctcgccaccagagccattccagagctcaccaaactgctgaacgacgaggaccaggtggtggtgaataaagctgcagtgatggtgcaccagctctccaagaaggaggcgtctcgccatgccatcatgcgctctccacagatggtgtcggccatcgtgaggaccatgcagaacaccaacgatgtggagacggcccgctgcaccgccggcacactgcacaacctgtcccaccacagagaggggctgctggccatcttcaagagcggagggatcccggccctcgtcaagatgctcgggtctcctgtggacagtgtgctgttttacgccatcaccaccctccacaacctcctgctgcatcaggaaggggccaagatggctgtgcgtctggctggaggtctgcagaagatggtggcgctgctcaacaaaaccaacgtcaagttcctcgccatcaccacagactgcctgcagatcctggcctacggcaaccaggagagcaagctgatcatcctggccagtggaggaccgcaggctctggtcaacatcatgagaacatacacctatgagaagctgctgtggaccaccagccgcgtgctgaaggtgctgtccgtctgctccagcaacaaacctgccatcgtggaggctggtggtatgcaggctcttggccttcacctgacagaccccagtcagcgtctggttcagaactgcctctggaccctcagaaacctgtcagatgctgccacaaagcaggagggcatggagggtctgctgggcacgctggtgcagctgctggcgtctgatgacattaacgtggtgacctgcgccgccggcatcctctccaacctcacctgcaacaactacaagaacaagatgatggtgtgtcaggtgggcggcatcgagtcgctggtgcggaccgtgctcagagcgggagaccgagaggacattacggagccggcggtgtgcgcactgcgacacctcacctccagacaccaggacgcagagatggcccagaatgcagtgcggctccactatggcctgccagtggtggtcaagctgctccacccgccctcacactggccgctcattaaggccactgtgggtctgatccggaacctggctctgtgtccggccaatcacgcgccgctccgcgagcagggcgccatccctcgcctggtgcagctgctggtgcgggcgcatcaggacacacagaggcgcacctccatgggcggcacacagcagcagttcgtggagggcgtgcgcatggaggagatcgtagagggctgcactggtgctcttcatatcctggccagagacgttcacaaccgcatcgtcatcagaggactcaacaccatccctctgttcgtacagctgctgtattcccccatcgagaacatccagcgtgtagcagcgggcgtcctctgtgagctggcgcaggataaggaggcggcggaggccatcgaggctgaaggagccacagctccactcactgaactgctgcactccaggaacgagggcgtggccacatacgccgcagccgtgctcttccgcatgtctgaggacaaaccacaggactataagaagcgtctgtcagtggagctcaccagctcactcttcagaaccgagcccatggcctggaatgagactgctgatctaggtctggatatcggtgctccaggagagacactcgcatacagagcagacgagcccagttaccgctccttccactcgggctatgctgcagaggctctgggcatggagggtctgctggagcaggagatgtcgcatcaccccggggcagagttccctgtggaggcgctgccggatctggcccacgctcaggatctgatggacggcctgcccaacactgactccaatcagctggcctggttcgacacggacctgtagagagctgctgagcgaaggacagctgaatgaagagagaatctgtatggtgaaggaaggtttgaggatgagctggaggttcagaaagtgcctgatgagtgcgtacacacacacacacacacacacacacacacacaggaggacagctggacggacggctgtagtgtagtccagatggagcagtgggacgcggttgcggacgaacacacggactctcggtctgaagacggaactcttttactctttttattttcatcctcttcggttattcctcctcctcctcctcatggtactgacccgagctttgtttttatcctgctcttttctttgtgtttgtgtagtgtttagtagcgtctctcctcatgggtcggtcactgatgtacaacactaatctgagttctgtctgatcgggccgaacgcagaatatcgagtagcttttctatgcggggttaaacggtggttattgagtacagaacctatattatatgcattgtggaggtgttctgggtgtgttctgggtgagtggggggggtagattactgggtcaaacacacacacacacacacacacacacacagatcttttctatgatgatatgaggagcgatggcgcttcttctgggaacacggaggaggacgaggctgatcaataagagactttattccatttgtccactggtgccatgctcagagtttcaggcctgctggatgttttctgtcgtcatattttagttgtgctttttaaaccgctatacgcatttatcttttacatttatttaggattcttcacctctttgttgttggtccagtagagctccattattcctgtataatttaagcctttttcttagttgtagacacactgtggtcagctctgtcagctccatgctggagtttataagacaacagtaaaggatattatgactttctacatgttgactttcttgtatctttaaaggtttctttccctcctgaataatgagggggaagtagagctggcagacgattaaccacatgcagaataaagtttgtatttacataaaaaaaaaa

***cx41.8***

cx41.8 2’OMe ASO#1 (GC = 55%)

mG*mC*mU*mU*mU*A*C*T*G*T*T*G*G*T*G*mA*mA*mG*mA*mG

cx41.8 2’OMe ASO#2 (GC = 35%)

mG*mA*mA*mU*mA*A*A*A*G*C*C*T*C*T*G*mC*mU*mA*mU*mU

cx41.8 2’OMe ASO#3 (GC = 65%)

mC*mC*mA*mC*mA*C*C*T*T*T*C*C*C*A*C*mC*mG*mA*mG*mG

>NM_001034988.1

gccgttcggcctgctggcagctgcttctgatcatcagcgtgtctgtaatctttcgtcccacagaagagacagtgagcaggattcatctgagggaagattgattttttgaggtcaccgtctagaaaaacaaacacaatcggcagttcattcatcaagtccggctttatcgttgaaggagacctttcattttgtcaagcggagtaataaaaagtcaatttttggcattaatctaaggatgatattgacaacgagtgatctgtctactacaattcttgagtccaaagttcaatgctttaagaaaaatatctttaatcctgctgaggtgtcaacaaacctgcttggtctcttcaccaacagtaaagcatcaacagtctgactctgtccattcaggatctctaacctcaggcattaatcgcacaagcgttcatcaaagggaagcttcagctggagatccgaagtcttggaggctctgtgactgtcagactgccaagtggttctggcaacaaatcgcacatcctgaaagcagagtagaggagaagagctttcgggtaaacaagagtctgtcaagtttcatcactaaatagcagaggcttttattcatcggtcaacagagatagaaacacacacctgtcagatctcaaacagacacgtgctgctacgctaactggtttcaatcaggctcctctgaataggc**ATG**gccgactggagtctgctggggagctttctagaagaagtccaggagcattcaacctcggtgggaaaggtgtggctcactattcttttcatcttccggatcttggtactaggcacggctgctgaatcctcgtggggcgacgagcaggaagacttcacctgcgacacagagcagcccggctgcgagaacgtttgttacgaccgagcctttcctatagcgcatatacgcttctgggtgctccagatcgtgttcgtgtctacaccttctctgatctacatggggcacgcaatgcatatcgtccgccgagaggagaagaagaggaaagagctggatgatgaaggagcgcagagagatggagaaaagtacccagaagatgacaagaacaaggaggacgaaggtggaggtaggagggtacgattgaagggtgcgttgctgcaaacatacgtcctcagcatcctcatccgcactgtgatggaagtgatcttcatcataatccagtacctgatctacggagtcttccttagtgcactctatgtgtgtaaagcccctccgtgtccacatccggtcaactgctacatctccagaccaacagagaagaacgtgttcattgtcttcatgctagcagtagcagcggtgtcactgctgcttagtatcgtggaactgtatcatttggcatggaagcagttgaggaagtatgtgcacggatacaaggcttccaaacaacgaccaaacacgccgtccaccatgcctgcactttcaccaaatccgtccaccccaaaccgagcctgcaccccacctccagacttcaaccaatgcttgacctcgccaccatcttctcctactttacagacacactcgcttttacatccgacctgccctccatttcacgaccgactggcgcaccagcagaactctgcaaacatggtcactgaaaggcacagaggacaagactacttaggggtcaacttcttgagcttctcacagacacctacagagactcccaactcctgtgcctcaccttcattcctgagcagtgattttgaggacaagcgaaggtttagtaagagcagcgggaccagcagccgcatgagaccggacgaccttgcggtatagcactggccagtcatatgtggaaattgggttgttctattgtgactaaaggctgatttatacttctgcatcaagcgcatgcgtatgctccggtgcaaccttcatgcagtcgaatagcccttgccgtggctaacgcctctcaaaaaaatgtaactacatgtcacaacgattcatagcgcaagctctgcgattggtcagcttggtagtgctgacaagcatggatggtgctgagagccgtgagccatatgcagcgattgcttatgtaaccccaccggtcctacaccacccaacccgctccgagctgggagcgaaccggcgaccttccgcatgggagtcggttgctctaacaaggaggctaaagaccatggcctctagtgtctgtcgctagagcacctttagaggtcagaggagtgaggattacctgcacagcacacaatagctggcctctgttacactcacccccctaaacctcactcccatccgggtcacggcaccaatgtaaccccaccggtcctacaccacccaacccgctccgagctgggatcgaaccggcgaccttccacatgggagtcggttgctctaacaaggaggctaaaaaccatggcctctagcgtccgctgctagagcacctttagaggtcagaggagtgaggtttacctgcacagcacacactagctggcctccgttacacttacagttgtcaagccctacgaaggagctccaaatggaaacttttgttttgtgtttaccttatgattaatgttgttgcacgtccgccggttcccgcctctgaataggcaagttttagctacgtattttaagaaactgaacacagaggaacatgcaaacatactgccagctagcgtgtcagaattgttattgcagagcaacacaaacagcacacagaagtatatatgcacgactacgcatcaaggcaagcaccgcgctgttcacgcccatcactcaatcgcagaagtataaaccagccttaagcctcaaaaaaaaaaaaaaaaaaaaaaaa

***dnd***

MO GCTGGGCATCCATGTCTCCGACCAT

dnd 2’OMe ASO#1 (GC = 40%)

mG*mG*mC*mG*mU*T*T*T*A*G*A*C*T*G*A*mU*mG*mU*mU*mU

dnd 2’OMe ASO#2 (GC = 55%)

mC*mG*mC*mG*mC*T*C*A*A*A*T*T*T*C*A*mG*mA*mC*mC*mC

dnd 2’OMe ASO#3 (GC = 55%): most efficient knockdown

mG*mC*mA*mU*mC*C*A*T*G*T*C*T*C*/iMe-dC/*G*mA*mC*mC*mA*mU

dnd 2’OMe ASO#4 (GC = 50%)

mA*mA*mC*mC*mA*T*C*T*C*A*T*C*T*C*T*mC*mU*mG*mC*mC

acaaacatcagtctaaaacgccttgtcttaaaacaccttaagtgaaatttcctctgagggtctgaaatttgagcgcgtttcacagaatccgcccacttgctccaacagtccaatagcgtctctaggagtcatgaggtttcttttaatgaccttttcttgacttttccaccaatttacaggtgtgtctatcatcatcatcacag**ATG**gtcggagacatggatgcccagcagcaggagcttcagcagattctgaacccgcagaaactcaagtctctgcaggaatggatgcagaggaactccatcactttaacccaagtcaatgggcagaggaaatatggtggtcctcctccaggttggcagggtcctgctcctggttcgggctgtgaggttttcatcagtcagatcccgaacgacgtgtacgaggaccgcctgatccctctcttccagagcatcggcaccatttacgagtttcgcctcatgatgaacttcagcgggcagacccggggcttcgcctatgctaagtacggtgaccctcttacagcctcggctgccgtcaccacgctgcatcagtaccggctgccggaggggggctgcctgaccgtgcgcaggagcaccgagaagcgccagctgcgtttgggggatctgcccgtcagcatgaatgaatcgaagctgctgatggtgctccagatgctctctgacggtgtagaggacgtcctgctcaagccaccggggcccaaagggaaagaggttgtggctctagtcaactacacgtcccattacgccgcatccatggctaagaaagtgctcgtggaagcttttcggaaccggtacggcatttccatcaccgtcagatggacttccttctccaagtccaagcgcgtcgaggacactccccaggaagacagctgcgtaaccccacttgttctgaagccgctttctaaaccatcactcctgcattatgacgtcccagctcaccagtctctgcttcctctcttccgggctgttgggggtccgaccaccagtgagcagagagatgagatgattcctcaacccaccataatgtcaagaaatgagctgattcctcaatcgtccataaggcagagagatgagatggttcctcagctccccataaggccgagagatgggatggctccccaatcccccattagtctcgacgccgtgtctcatctgcagtggatgtgcgaggtcaacagactcggctctccgcaatatgaagtccacttccatcacgcggctcctgacggattcctctacttcgccttcaaagtgctgatcccaggcctgccgctgcccctgtatgggttcgtccagatcctgccaggcaccagcgcacgagccatgaagagtgaagtttaccgggccgccgctgagcaggtgatccaaaccttgtgccgagtctcaaatttacggcctttctaagaatgtcagattatggcttgatcgaatgtgattgtgatcagttttacgttcagtattatgtactgttccggttatagatgatgaatatgtggaaatgtaatgaaaaataagcatttagtttactgttgatgaagagaaaaaaaaaaggtgaccaaggcagtattacttttatttgattttatttttttcaagctcttgaatttagtggtttgaagttttatgttctcgtcgttttataatattttaactatgtaatattaataattgagttgttttagtcagcctcatcatattaggatgactgcatgttttcacgcttttcttttgagtgtttttcactgtatttcgacttcactttggtttgcgtttgtcacgattgttctttttgcatggtgtgctccttgtgtttccttgtttgatgggttgtactgactataaatgacttttgtacaataaataagttgtt

***hcrt***

hcrt 2’OMe ASO#1 (GC = 50%)

mG*mU*mC*mC*mC*C*T*G*T*C*T*T*G*T*A*mG*mA*mC*mA*mA

hcrt 2’OMe ASO#2 (GC = 50%)

mC*mU*mU*mA*mG*C*T*G*T*G*C*A*G*T*C*mC*mA*mU*mG*mA

hcrt 2’OMe ASO#3 (GC = 60%)

mG*mC*mC*mA*mU*G*A*A*G*A*/iMe-dC/*G*A*G*C*mA*mC*mC*mU*mG

>NM_001077392.1

gaaaatcaagacttttcgatacatgacatttgtctacaagacaggggacagaagctaaactc**ATG**gactgcacagctaagaagctccaggtgctcgtcttcatggcgctgctagctcacctggccagggacgcggaaggcgtggcctcctgctgcgcgcgcgctcccggctcctgcaaactctacgagatgctgtgccgagcaggacgcagaaacgactcttccgtcgccagacatttagtgcatctcaacaacgacgccgctgtcgggattctcactcttggcaaacgtaaagtgggcgaaagccgcgtccacgaccgcctgcaacaactgctgcacaactcacggaatcaagccgcggggatcctcacaatggggaagaggctggaggagcccgctaaattcctcatccctacggtaccacaggatgtggacagttatgaaaaacgatgacacttgtcgctatttaataaaaaaaaaaaaaaaaa

***mitfa/nacre***

MO CATGTTCAACTATGTGTTAGCTTCA

Mitfa 2’OMe ASO#1 (GC = 40%)

mC*mU*mC*mC*mA*A*C*A*T*G*T*T*C*A*A*mC*mU*mA*mU*mG

Mitfa 2’OMe ASO#2 (GC = 45%)

mU*mG*mA*mU*mG*T*G*C*A*A*G*A*A*C*T*mG*mA*mC*mC*mA

>NM_130923.1 gccaagacgactggtcagttcttgcacatcacctaaattcacaaagggaatacttttttgaagctaacacatagttgaac**ATG**ttggagatgctcgagtacagtcactaccaggttcagacccacctggaaaccccctcgaagtaccacatccagcagagccagaggcagcaggtgaagcactacctgtccagtgcactgggagccaagctgagcccacaggccagcacagggcccggccccagccagcccgccgagcacggcatgaccccgggacccggagccagcgctcccaacagccctatggcccttctcaccctcaactgtgagaaagagatggacgatgtaattgaagacattataagtttggaatcaagctacagtgatgacattcttgggttcatggatgcaggacttcaaatgacaaacacgattccagtttcagcaaacctgctggacatgtacagcaatcatgctcttcctccagctggagtttccatcagtaactcctgcccctcaagcctgccggccgtcaaaagggaattatccgttactccatctccgggcatgatgcacattatggacaaagctggaccatgtggcaagtttgactcttatcaaagacctgatggctttccagtagaagcagaagtcagagccctggcaaaagagagacaaaagaaggacaaccacaacctcattgaacgaagaaggcggtttaatatcaatgacagaattaaggagctggggactttaattcccaagtcaaatgatccagacatgaggtggaataaaggcaccatcctgaaagcatcagtggattacattaggaaattgcagaaagagcagcagaaagcaaaagagctggaaaacagacagaagagactagaacacgcaaacagacatctcctgctccgtattcaggaacttgaaatgcaagcccgtgctcatggactcaccgttgtagcttcttccagcctttattccgctgagttagtggctcgagcaataaaacaagagcctgggatgggggactgcacatccaatttgtacccacaccttcccagtcctgacatgtcccgtcctaccactctggacctcaacaacggcaccatcagctacaatgacagtcccacggaggacggcgagccgggggtctacgacagcccaaacaaggcctccaccaagctggaggacatgcttatggacaacaccctgtctccggtgggcagcagcgacccgctgttgtcctccggatccccagtaccttccaacagtagcggcagcagcagtatggacgaacatgacaatggctgttagcaatgccaactaaatttcatgaaccaaagttagacaattagatttgtgtgtgtgtgtgtcttgtaagtaccagtgttacatccagatcatgaagagcgcaatttcgtaaagtattaatcttgtatttcagaatatttagtgtgcttgtgtatatattttttcatttatcatgtcagagtctagtcaagaatgaaaacgtgtaaacaattagttgatttttgaaggattctcgcggatgatataaagaggagcttgaataaaaaaaaaaaaaaaaaa

***oep***

Oep 2’OMe ASO#1 (GC = 55%)

mC*mA*mU*mU*mC*T*G*A*C*C*C*C*T*C*A*mC*mA*mU*mC*mC

Oep 2’OMe ASO#2 (GC = 45%): most efficient knockdown; used for rescue experiments

mG*mG*mC*mG*mA*A*C*A*T*G*A*C*A*A*T*mU*mG*mU*mA*mG

Oep 2’OMe ASO#3 (GC = 45%)

mG*mA*mU*mC*mA*T*T*T*T*C*T*T*T*G*T*mU*mU*mG*mG*mG

Oep 2’OMe site1_5’UTR (GC = 40%)

mC*mA*mA*mA*mA*C*A*A*C*T*/iMe-dC/*G*A*T*T*mG*mA*mG*mG*mT

Oep 2’OMe site2_5’UTR (GC = 45%)

mT*mC*mA*mT*mT*C*/iMe-dC/*G*C*T*G*G*C*C*A*A*T*A*A*A

(ORF in upper case)

gtgagatggagatgttctaatggtgtttttgggatacgaaaacttacctcaatcgagttgttttggagtttattggccagcgga**ATG**ACGAGTCAACTGTTCGGGTTCTTGATGTTTGCTGTGATTATTTGTCAAGCTGTTTCACTCGAGTCAGGATGTGAGGGGTCAGAATGTGTGAAAGTTGGGGTTTCTGGAAAACCAAAGCAATACGCTGAATTCTTGAATAAATTTAACGAAATGAACACGCAAACGCCGCAACGTCAACACCGCAACGCCGAGGCAGCGTTACCGTTCGTTGGACTGACCGGAGTTGCCAAACAAAGCCGTACCTGCTGCAAGAATGGGGGAACGTGCATTTTGGGAAGTTTCTGCGCCTGTCCGAAGTACTTCACCGGCAGGAGCTGTGAATACGATGAACGCCTCAGGGATTGTGGTGTTATTCCACATGGAGAATGGGTTCAGAAAGGATGTTCGTACTGCAGATGTGGATATGGACTTTTGCATTGCTTCCCACATGTTTTCAGCAAAGACTGTGATGTTTTCAGCAAAGACTGTGATGACTCTCAGGAAGTTCGGTGGCACCGGTCGGGCTCCCTCAGAACACTGTCGTCTACAATTGTCATGTTCGCCACTTTTATTTTACACCGCCTGCTGTAAatgagttttcctaaagggatagttcacccaaacaaagaaaatgatctcccctgttgaacgcagaagaagatactttgaaaaatgctggcacttgacttccatagtaggggaaacattactatggaagtgaaccagcatttctcttttgtgcccaagagaaggaagcagctctcaaataggttttaaaccgttgaaggatgagtaaatgtgttcatttttgtgcgaactatccctttaacaaacagaaagcggttttattttagattttttctgtctgacaggttcgggca

***slc24a5/golden***

slc24a5 2’OMe ASO#1 (GC = 45%)

mC*mA*mU*mC*mA*T*T*C*A*G*C*A*G*A*A*mC*mA*mC*mA*mG

slc24a5 2’OMe ASO#2 (GC = 45%)

mC*mA*mC*mA*mG*T*A*C*A*G*C*T*A*C*A*mU*mA*mC*mA*mC

slc24a5 2’OMe ASO#3 (GC = 50%)

mU*mG*mU*mA*mA*C*A*/iMe-dC/*G*C*C*A*A*A*C*mA*mC*mA*mC*mC

>NM_001030280.1

gtaagccgcggcggtgtgtgtgtgtgtgtgtgttctccgtcatctgtgttctgctga**ATG**atgaggacagacgtgtttctccagcggaggaagcgtagagatgttctgctctccatcatcgctcttcttctgctcattttcgccatcgttcatctcgtcttctgcgctggactgagtttccagggttcgagttctgctcgcgtccgccgagacctcgagaatgcgagtgagtgtgtgcagccacagtcgtctgagtttcccgaaggattcttcacggtgcaggagaggaaagatggaggaatcctgatttacttcatgatcatcttctacatgctgctgtccgtctccatcgtgtgtgatgaatattttctgccatctctggaggtcatcagcgagcgtcttggtctctcgcaggatgttgctggagccacgtttatggctgcggggagttcggctccagagctcgtcactgcatttctgggtgtgtttgtgacgaagggcgacatcggcgtcagcaccatcatgggttctgctgtctataacctgctgtgcatctgtgcagcgtgcggcctgctgtcctctgcagttggtcgtctgagctgctggccgttgttcagagattgtgttgcgtacgccatcagtgtcgccgccgtcatcgccatcatctcagataacagagtttactggtatgatggcgcgtgtctcctgctggtgtacggtgtgtatgtagctgtactgtgtttcgatctgaggatcagcgagtacgtgatgcagcgcttcagtccatgctgctggtgtctgaaacctcgcgatcgtgactcaggcgagcagcagcctctagtgggctggagtgacgacagcagcctgcgggtccagcgccgttccagaaatgacagcggaatattccaggatgattctggatattcacatctatcgctcagcctgcacggactcaacgaaatcagcgacgagcacaagagtgttttctccatgccggatcacgatctgaagcgaatcctgtgggttttgtctcttccggtcagcactctgctgtttgtgagcgttcccgactgcaggagacccttctggaagaacttctacatgctgaccttcctgatgtccgccgtctggatttctgcattcacttatgtgctggtctggatggtcacaatcgtgggggagactctgggaatcccggacacagtgatgggaatgactcttctggctgcaggaaccagtatccccgacaccgtggccagtgtgatggtggcccgagaaggtaaatctgatatggccatgtccaacatcgtgggctcgaacgtgttcgatatgctgtgtctgggcctgccgtggttcatccagacggtgtttgtggacgtgggctccccggtggaggtcaacagctcggggctggtcttcatgtcctgcacgctgctgctctccatcatcttcctcttcctcgccgtgcacatcaacggctggaagctgaactggaagctgggtctggtgtgtttggcgtgttacattctgttcgcaacactctccatcctgtacgagctcggcatcatcgggaacaatcccatacgctcctgcagggactgaacactgctctacagcgcccccttatggacaacacaaggatgtgactctttataaccctctaaagtgcacaggttcattactgaatacaagaaaatagaactgcgagacgtcaactcaaaatacaagagaagtcaaagtgcgagatgtaaaaaatatatgcacataaatgaggataaactttttatttaatatgacaaaactgcataaagtctgatgtgaacactgctcaacagcgccctctcatggacaacacatggatctgactcttattaaccctccagagtgcaaatacactaacacaacgtaatataaccaagttaaaatggcaagatgtgaactcaaaatacaagaaagcagtcaagatgcccgacataacaaatgtgcattaaaatgtaagccct

***smad2***

smad2 2’OMe ASO#1 (GC = 60%): used for knockdown experiment

mU*mC*mC*mU*mA*A*C*T*G*G*G*T*C*T*C*mU*mG*mG*mG*mC

smad2 2’OMe ASO#2 (GC = 65%)

mC*mA*mC*mC*mA*C*C*T*C*C*T*G*C*T*C*mC*mG*mC*mU*mU

smad2 2’OMe ASO#3 (GC = 60%): also produces efficient knockdown

*mU*mG*mG*mC*mC*G*T*T*T*T*G*C*T*C*T*mC*mC*mA*mC*mC*

>NM_131366.2

gtcgtctgctgctgtaattcggctacagtgggaaggaaaatattgttttaaaacgacttttacccactcgcgaataatcagtttcgcacggattttacgccaccgctttattacggagaacgcttttcgtaggaagggtaacccctcacccccctttcctctcctgtttcctaaacatctatgccccctgccccctcataatcagcccagagacccagttaggaactttgggtctgttcctccccattctcctagcctccatctgtctctctaactccacctgcccagactgaccgctcatcggacaccccaagaaaaaaaaaaac**ATG**tcctccatcttgcctttcactccgcccgtggtgaaacgtctgctgggttggaagaagtctgctagtggttcaagcggagcaggaggt*ggtggagagcaaaacggcca*ggaggagaagtggtgcgagaaggcagtcaaaagcctggtaaagaagttgaagaaaactggccaactggacgagctggagaaggccatcaccacacaaaatcgcaacaccaagtgtgtcaccattcccagcaattgctctgaaatttggggactgagtacaccaaatacgatagaacagtgggatacctcaggcctttacagctaccctgaccaaaccagatctctggacgggcgtttgcaagtgtctcaccgtaaaggtctgcctcatgtcatctactgccgcctgtggcgatggcccgacctgcacagccatcacgagctgcgcgccatcgagacctgcgagtatgctttcaacctcaagaaggatgaagtctgcgtcaatccctaccactaccagcgggtggagacacaagttcttcctcctgttctcgtgccaagacacacggagatcctgactgagttgccacctttggacgactacaccaactccatacctgaaaacaccaacttcccaacagggatcgagccccccaacaattacataccagaaactcctccaccgggatacatcagtgaggatggggaggccagtgaccagcaaatgaatcaaagtatggacacaggttctcctgcagaactgtcaccaagcacactctctcctgtcaatcatggcatggacctgcagccagtgacttactcagagcctgcgttttggtgctccatagcttactatgaacttaaccagcgggtcggagaaacattccacgcctctcagccttccctcaccgtggacggcttcacagacccctccaattcagagcgattctgcctgggcttgctgtccaacgtcaaccgcaacgccaccgtcgagatgacccgaagacatataggacgaggagtcaggctgtattatattggtggggaagtgtttgctgaatgtcttagcgatagcgccatctttgttcagagcccgaactgtaatcagaggtatggctggcatcctgcaacagtctgtaaaattcccccaggctgtaacctgaagatcttcaacaaccaggagtttgcagcgttgctggctcagtcggtgaaccagggcttcgaggctgtttatcagttgaccaggatgtgcaccattcgcatgagttttgtcaaaggctggggagcagagtacagacggcagacagtgacgagcaccccctgctggatcgagctgcatctgaacgggcccctccagtggctggacaaggttctgacccagatgggctccccctccgtacgctgctccagtatgtcctaaacccagctctctgtcctctgcccaccccaaaacaatcacatcaagcgacaactcgaagaacaacaggcttacgatgcccccctcgctcctttctctttcatagtactcgtgagcctttcttctatcctcttctctatcctctcgacactgatgttcggacccgaacgcagcacttgctgtctcatcagcttcatgcacctttgattctctcttgttttctttcataaaccctcaatattaaatgtattagtagtagaaaaaaaaaaaaaaaaaa

***smad5***

MO AACAGACTAGACATGGAGGTCATAG

Smad5 2’OMe ASO#1 (GC = 45%)

mG*mA*mC*mU*mA*G*A*C*A*T*G*G*A*G*G*mU*mC*mA*mU*mA

Smad5 2’OMe ASO#2 (GC = 45%)

mA*mU*mG*mC*mC*A*G*A*T*A*T*T*G*G*T*mU*mG*mU*mG*mG

>NM_131368.2

acttcgcctccagcctgtttgacgccaggagggtgagagacgaggggaagagaggcaggaaaaaaggaaaaaaactccttcatgtcggtgtctgtaaaacagagcaaaggtcgacagagtctacagcatttcttgaaagggatggtggcttttccgcagggaggtgttgagaagtgattgaaaaagtttccttgctgttttttttctcctttttgtgtgaatgaggtggactttgagtccggtgtcagtctctgaccacaaccaatatctggcatggattagtttataaaatctcctaactgcctggttgtgtgtttccagccttgattcctcaattgccctttacgctaattctcgcagtagttgtgacccagttcctcccccggcttcactgcaggccttcctgagccccaagtaccagcagctgcgtcctgctttccacttcctgtccttggtcctgcaaggctaagcctgtccacttcccccctccccccctgacatacacaaacacacacataatcatcttcctggcacactgctggccgaggacgctccagattcggcttcctggtgcagcccagcact**ATG**acctccatgtctagtctgttttccttcaccagcccggcagtgaagcggttgctgggctggaagcagggcgatgaggaggaaaaatgggcggaaaaagctgtggatgccctggtgaagaagctgaaaaagaaaaagggtgccatggaagacttggaaaaggcccttagtagtcctgggcagcccagcaaatatgttaccattccccggtcgctggacgggcggctgcaggtgtcccacaggaagggcctcccacacgtcatctactgccgcgtgtggcgctggcctgacctgcagtcccaccatgagctcaaaccgctcgaggtttgcgagtatccattcggctccaaacagaaagaagtgtgcatcaacccatatcactacaaacgagttgaaagtccagtacttcctccggttctggtgccacgtcacagtgagttcaaccctcaacacagtctcctggtgcagtttcgcaacttgagtcacaacgagcctcatatgcctctcaacgccaccttcccagagtccttccagcagcacagcggaggaagctccttccccatctctccaaactcaccctaccctccatctcccgccagcagtggcacataccccaactctcctgcaagctctgggccatccagccccttccagctaccagctgatactcctcctcctgcctacatgcctccagatgaacagatggggcaagacggttctcagtccatggagactggtagcagcctggctcctcagaacatgcccagaggggatgtgcagccagtggagtatcaggagcccagtcactggtgctctattgtgtactatgagctgaataatcgtgtgggagaggcttaccacgcttcctccaccagcgtactggttgatggattcaccgatccatccaacaacaaaaatcgcttctgcctgggcctgctctctaatgtcaatcgcaactccactattgaaaacacccgtcgccacatcggcaaaggtgtccacctgtactatgttggaggagaggtgtatgcagagtgtttgagtgataccagcatttttgtccagagtcgaaactgcaactaccatcatggctttcaccccacgactgtctgcaaaatccccagcggctgcagcctcaagatcttcaacaaccaagaatttgctcagcttctcgctcagtctgtcaatcatggctttgaggccgtctatgagcttaccaagatgtgcaccatccgcatgagctttgtaaagggttggggtgccgagtaccacagacaagacgtgacaagcaccccctgctggatagaagtgcatctccacggccccctgcaatggctggataaagttctaacacaaatgggttcccctctgaaccccatctcttctgtctcgtaatgatgggctgacctgggagaagcctttgttttcttcttcttattttctttcttcacttttgaaagaataggaggtcttaagggctgaactgtttacacaacctggagaatgccaccaaatcctgcagcagtcagaacgagaagatgaactggggctacaagcacatctagcataggaaaattctaggcagtgacacaaaatggactaactactgctttagcaattgacttcatgttttgttttgttgttttttttgcatacagttgttttacaatgatgcttgtatgactgattttctctaactgtaggtaatctagtagatgacattgacatgctttcatgtatctctacttcactttacaccagttgaaaaatgcaattgcagtttcctagttctatgagaatctttttaattgctatgttgtctaatcctgttattcgcaaggcatattttcctttttatgtttactgttagactcaaagctcattacaaatgttccctttttatagcatctgttgcctttaggcaaatcaaagttactgcttttaaataaagttcttttatctgatgaaaaaaaaaaaaaaaaaaaaa

***ta-T/ntla***

MO GACTTGAGGCAGaCATATTTCCGAT

Ntla 2’OMe ASO#1 (GC = 45%)

mC*mU*mU*mG*mA*G*G*C*A*G*A*C*A*T*A*mU*mU*mU*mC*mC

Ntla 2’OMe ASO#2 (GC = 50%)

mG*mA*mU*mA*mA*G*T*C*/iMe-dC/*G*A*/iMe-dC/*G*A*T*mC*mC*mU*mA*mC

>NM_131162.1 gaattcccgctgtcaaagcaacagtatccaacgggatttagtaggatcgtcggacttatctcaagctttatttgatcggaaat**ATG**tctgcctcaagtcccgaccagcgcctggatcatctccttagcgccgtggagagcgaatttcagaagggcagcgagaaaggggacgcgtccgagcgggatattaaactttcgcttgaagacgcggagttgtggaccaaatttaaagagctcaccaatgaaatgattgtcaccaagactgggagacgaatgtttcccgtgctcagagccagtgtcaccggtctcgaccctaatgcaatgtactcggtcctgctggattttgtggcggccgataataatcggtggaaatacgtgaacggtgaatgggtgcccggtgggaaacccgaaccccaaagcccgagctgcgtctacatccacccggactcacccaacttcggcgcgcactggatgaaagcacccgtatctttcagcaaagtcaaactctccaataaactcaacggaggaggacagattatgttaaactcattgcacaaatacgaacccaggatacacatcgtgaaagtcggtgggattcagaaaatgatcagcagtcagtcttttcctgagacacagtttattgcagtcacagcatatcagaatgaagagattaccgctctgaaaatcaaacacaatccttttgccaaagctttcctcgatgccaaagagagaagtgaccacaaggaagtcccagaccacagcactgacaaccagcaatctggatattcacaactcggtggctggttcctgcccagtaacggccccatgggccccagcagcagccctcctcagttcaatggggcccctgttcactcctcgggttcgtactgtgagagatactccagcttgaggaaccacagagctgctccatatcccagccattactcccaccgcagcactaccaccaataactacatggacaactcttccggaagtcttgcgtctcatgacagctggtcagccctgcagatccccaactccagcgggatgggaaccctggcccacaccacaaacactacctccaacaccagtcagtacccaagtctgtggtcagttgcagggacgactctcaccccatcaggctcagcatcgggctccattacaggtggcctgacatctcagttcctacgcggttcttcgatgtcctactcgggtctgacctcctcgctgcctgtgtcctctccctcctcaatgtacgatccaggcctaagcgaggttggcgttggagatgcccagttcgagagctccatcgcccggctcacagcatcatgggcgcctgtggctcagagctactgagatcgcttcacatttaaggactgatgctgcagttatggacttgatcttggcttcaggaggaaatctagaagagcttcttgatttgacaatcagaaaacgggttgatttactataaaagtcacatctgtatcataccgaggcatacgtatttacaatcaagatgagagacaatcaattaaagggttagttcttgcaaaaaagaaaattttgacatcatttactcacctttgttttaaacattgttaagtttttattctgttaaacacaaaagaagatattttgaagaatgttcaaaactggtaaccattgcatagaagctgttttacttatggaagtaaatggttacaggttatcagcatttttttaaatatattttttagttcaacagaagaaagaaactctttaaagtttggaacaacttgagggtgagtaaattgagtaaaagtacgtttttgggttaactatccctttaactatcagattttagccatacattttggggcaattatagtgtttattcttgataatattatctaaaagattaataaaatcaaaattgtgctgttgactcactaaaagtgtatatgtgtgtaaataaatagaaattaacgtccggtttcattgtatcacagaagaatgtaacagtcttacatgtgctttctgtagaacgagagaaagacagactttgctgtttcgtttgagaaagtgaatacgctttgaaaagtgaccgtatagttttgtctgctattcgtcctatagagaaaccatttgtacatatctatctatttgtatttgttgggctctttgagttttatttatgtcattttaataataaattaaatttcttttttttttctgtcaaaaaaaaggagttccggaattc

***toddler/apela/ELABELA***

toddler 2’OMe ASO#1 (GC = 40%)

mG*mA*mG*mU*mU*A*T*A*T*A*G*A*A*G*A*mC*mA*mC*mG*mC

toddler 2’OMe ASO#2 (GC = 45%)

mU*mU*mG*mA*mG*T*G*A*T*G*A*G*T*T*T*mG*mC*mA*mG*mC

>Zv9_00001691 (Pauli et al., 2012; Pauli et al., 2014)

cctcccctttctcttgtctgtataaagagggggtgaaactacattgtctcaactgaacattcccactcatcaggtcatctgtctatctatccatccctcagaggacagagagagaagagagagtgaatatcgccctctcaaactttgaaaaagttggagagaccgagagctggctacaactgcgtgtcttctatataactcaacttatatgagatctgagaacacttgctgagagcgacagacacataagaggatttctacagtccgttacctgcacatccgacagaatttatcgtctgaggaaccgcggacatcctgtgaggagagtcgagtctgcgccgcggaccaaaccaccctgagcatcacc**ATG**agattcttccacccgctgtatctgctgctgctgctgctgacagtgctggtcctcatcagcgcagataaacatggtacaaaacacgattttctcaacttgaggcggaaatatcgcagacacaactgcccgaagaaacgctgtctacctcttcactccagagtacctttcccttgaggttttatgatgctccgggcaagcattaagaaaaaccaaagaccagccttggattggaaatgagagaagatttatgtcagatgtgccgaggactgttttattcgcacatgtattgtaatcaaagccatgtttgtctcttctgtagcagaagtgttttttgttttgttttgttttttaaatgaatgtaagtgaatgagccatggagatcctactgctgccaaacatgctgcaaactcatcactcaatcaggttgagttggagcagaatcattgtaaatagtaaggactgaatgaaatgtgtttatatgtaagttatgcacttcaaatgttttattattatcttgatttattaaaagtgtattgtcttttcagatgggtttagtgtgccttattgatttactacttgccatagaacatagctatagaatttacaaagtggcaacatggtggctcagtggttagcactgtcgcctcacagcaagaaggtcgctatcccggctgggtcagttggcatttctgtgtggagtttgcatgttctcccagtgttagtgtgggttttcttcaggtgctctagttttctccagagtcaaaagacaagcagtacaggtgaattgaataagctaaagtaaagtttcacaaactaatttccagagggac

***tolloid/mini-fin***

MO GCAGAGTAAAGGTAGTCCATCTGAG

tolloid 2’OMe ASO#1 (GC = 45%)

mC*mA*mG*mA*mG*T*A*A*A*G*G*T*A*G*T*mC*mC*mA*mU*mC

tolloid 2’OMe ASO#2 (GC = 50%)

mA*mG*mA*mG*mG*A*T*A*C*C*A*G*C*T*C*mA*mC*mU*mC*mU

>NM_131010.1

cgttaatctctagggaaatgggcacgtttggaataagcggtaccagtcagctagcgcactctaatattccagtggctgtagaaaaagtgtttccagagagagagagaagagagagggagggagagagagggaatatgagggcagtactcaggatgagcctgcgctttgaggctgtaaaacactagaccctgagggcattctctgtctgagctgtgcctcttcaggaaaactgactgcgcaagagaaaggaagagtgagctggtatcctctgtagttgtaggttcatattgatgcatttggcgtccagctgagatgccgcgacgctgacggtcctgccccgtctgtcgaaccggaccaatgatgataatgaaggcgatgatgtccacacggcgcgtgtagccgcctaaattccacactgccggactcctaccagacactccttctgacccacgctcgttcacacctcag**ATG**gactacctttactctgcactcaccagtaaaatgaattggatcgcactcctgctggccggcttgactttttgttgcaaagtgtccgtgcacagctgtttagactacgatgacagttatgattattacgaggaggagaaaacagagacgatagactacaaggatccctgcaaagcagctgtattttggggagacattgccttggatgatgaggacttgaaaatgtttcacatcgatgggacgatagaccttaagcaacaaacacatgggaggcaaggacacacatctggaggtctaggagaacatgtgcccactaagaagaggggttccttatatctgctgctagacagaatacgacggttaggttttgagtcgtggccagtaaacagcagtaaagatgtgtcaagcataaagactggaataaggagagtaaacagcgccagaaatgtaaagtctcgagtcccacgtgctgctacatcccgagctgagaagatctggcctggaggagtcatcccttacgtcataggaggcaacttcaccggaagtcagagggccatgttaaagcaagcaatgagacactgggagaaacagacgtgcgtgactttcattgagaagactgatgaggagagctacattgtcttcacgtacagaccttgcgggtgttgctcttatgtcggccgccgtggaaatggtccccaggcaatatctatagggaaaaactgtgacaagtttggcattgtggttcatgaacttgggcatgtgatcggcttttggcatgaacacacgcgacctgaccgtgacgatcatgtgaccatcatccgggacaacatccagccaggtcaggagtataacttcatcaagatggaaccaggggatgtcaactctcttggtgagccgtatgattttgacagcatcatgcattatgccagaaacactttctccagaggaatgtttttggacacgattcttccctctcgtgacgagaatggcgtcaggcctgctattggtcagagaaccaggctcagtaaaggggatatatcgcaagccaagaagctgtacagatgcccagcatgtggcgaaacactacaggactcagtggggaatttctcatctccaggatatcctaatggatacccatcatatacacactgtgtatggaggatctctgtcacacctggggagaagatagtgttaaacttcaccactatggacctctacaagagcagcctgtgctggtatgactacattgaggttcgtgacggatactggagaaaagcgccattgctgggccggttctgcggtgataaaattccagaagttctggtctctacagacagtcggatgtggattgagtttcgaagcagcagcaactgggttggaaagggatttgcagcagtctatgaagcaatatgtggaggggagatcagcaaggactctggacagattcagtctccaaactatccagatgactatcgcccatctaaggagtgtgtgtggaggatcacagtgtctgagggctacagcgtgggcttaagctttcaggtttttgagatcgagaggcatgacagctgtgcatacgactatttggaggttagagatggattgtcagagaacagccctctgattggtcgattctgcggctatgataaacctgaagatattcgttctacctctaacaacctctggatgaaatttgtctctgatgggactgttaataaagcaggctttgctgcaaacttcttcaaagaggaagacgagtgtctgaagccagataatggaggctgtgaacagagatgtgtgaacacattaggaagcttcaaatgtgcatgtgatcctggatatgaactggctcctgacaagaagagctgtgaagctgcgtgtggcggtttgttgactaagttgaatggcacaattaccaccccaggctggcctaaggaataccctcccaacaaaaactgtgtgtggcaagtagtggccccgactcagtaccgtatatccatgcagtttgaagcatttgagctggagggaaatgaggtgtgcaagtatgactatgtcgaggtgcggagcggcttgtcatctgactcaaaacttcatgggaaatactgcggcacggaagttcctgaggtcatcacctcccagtacaacaacatgcgaatcgagttcaaatcagacaacacagtctccaagaaaggcttcaaagctcatttcttctccgataaagacgaatgttcaaaggataacggtggatgccagcatgagtgcatcaacactattggcagctatgtgtgccagtgccgcaacggcttcatcctacatgagaacaaacatgactgcaaggaagctgagtgtgagcacaaaatccacagcacgactggaaccatcagcagtccaaactggcctgacaaataccccagcagaaaggagtgcacgtgggacatcaccgcaacccctggtcaccgggtcaaaatttcttttaatgaatttgagattgagcagcaccaggaatgcgcatatgatcacctggaggcctttgacggcgattcagacaagactcctatactgagtcgcctgtgtggcaataagattcccgaaccactcatttccactggcaacaagatgtatctgcgtttcatctccgatgcctcagttcaacggaaaggctttcaggccactcattccaccgaatgtggcggaaggctgaaagcagaagcacgacagaagaacctgtattctcatgctcagtttggagataataactaccccggacacaccgactgcgagtggctcatagtggcagagtcgggttatggcatcgaactcaccttcaccacctttgaggtagaagaagaggcagactgtgggtacgactacatcgaactttacgatggctacgacactggagcgcacaaaatcggacgcttctgtggatctgggcctcgtgaggagctttactctgctggcgatgctgtgttgatccattttcactctgacgacaccatcagtaagaaaggctttcacatccgctacactagcacaaagtttcaggaggcgctacacacacgcaagtaacatttgagagactgaagacagggataaaatgaggagaaaaagactccagcaaactattgcaattgattcatttcaatcaaagaccctgccgttatgtgtttcctgcattggatttgcctctatgttcagccactatggaagtgctgtcatattgaccctaaacaccaatcagctgctcttatgtgtgggattgctccgcccactggagtggtgtcttgcgctctctgactggagcagtgcagtgaaaacacacacacacaatcacacacaaaactttcttgttggctctccattcttcagacatcgagttgcacactgaaactaagagacactgataataagacatttaagtaactgctattgaacgggcctgtgtgtatgtgtgtgtgttgctccggacagcatggacagtttggctgctgacgccacaccacactgtacctcagctaaaaatgtgtgtgtgtatttggatgtgtgtgtttgagagaaaaacagaaagagagagagagagagccttcaaataccagcctgaactcattcaaatgttttttttattgtggttgtacaattcttctgttattgttctgttagctttacggcaaaacccattttctctccatcaaaagtgcaaaagtaaatatgtagccctatttaacaataagcttccttacatgtatgtagcccattgtttatgtcagcggagacagacatcatttgtcagtgattaacattgagctggagggtctcattcactaagtatctgcatgcatcaaccggtgagggaaatttcaaccccatttctgaaaatgtacctttatataacatttctggagagcgccaaatacgtcccaggagctacgtttttgtcagtttttgtttttgcgaatccccagaggctgctgtgtatgctttttgagatcttaaatttctctcacatgccattcacacctgctgttctcttgtaaatccaccagagcccgctgttgactgactttctggctgaccaatcaacttgacccaccctcctcctttcctaaacctaaccaatagtattttaaaaagcacagattgacctgcccacccacttccctaaacg

***wnt11***

MO GAAAGTTCCTGTATTCTGTCATGTC

Wnt11 2’OMe ASO#1 (GC = 40%)

mG*mU*mU*mC*mC*T*G*T*A*T*T*C*T*G*T*mC*mA*mU*mG*mU

Wnt11 2’OMe ASO#2 (GC = 45%)

mU*mC*mC*mA*mC*A*A*/iMe-dC/*G*G*T*C*A*A*G*mA*mU*mA*mC*mA

>NM_001144804.1 tcagacagtccgtggtgtatcttgaccgttgtggaaaaacttcactggagtttctaaacagcaaaagagcgac**ATG**acagaatacaggaactttcttctgcttttcatcacgtcactgagcgtcatttatccatgcacaggaatatcatggcttggtttgacgataaacgggagctcggtgggctggaatcagacgcaccactgtaaacttctggacgggctcgttcccgatcagcagcagctctgcaagcgcaacctcgagctcatgcacagcattgtacgcgcggccagactcaccaagagcgcgtgcacgagctccttcagtgatatgcgctggaactgctcgtccatcgagagcgcgccacacttcacccctgacctggccaaagggacccgtgaggcagcgtttgtgttttctctggctgctgcggtggtcagtcatgccatagctcgtgcctgtgcatctggagacctgcccagctgttcctgtgctgcaatgccgtcagagcaggcggctcctgatttccgctggggtggatgtggagataaccttcgctacggcctacagatgggctccgctttctcagatgcaccaataaggaaccggcgctcgggcccacaggcctttagactcatgcagcttcacaacaatgctgttggcagacaggtgcttatggactctctagagatgaagtgcaaatgtcatggcgtttctggctcatgctctgtaaagacctgttggaagggtcttcaagacatcagcaccatctccgccgacctcaagtctaaatacctgtcggccaccaaggtgattccgcgtcagattggcacacgccggcagcttgtgccccgagagatggaggtgaggccggttggagagaatgaactagtctacctggtcagctcaccggattactgcacacagaacgccaaacaggggtcactggggaccacagacaggcagtgtaacaagacggcgagcggcagtgagagctgtgggctgatgtgttgtggacggggttataatgcctacacagaggtgctggtggagcgctgccagtgtaaataccactggtgctgttacgtgtcctgcaaaacctgcaagcgcaccgtcgagagatacgtctgcaagtgacagaccgtcttcaccaatagaccttgtgtgaccctttttttttagagccataagtgagtggataatcctttcccaggcaggaaagtggagaaagaagagccggagttgaggaatatgggatggtgaaagagttaatgaatggctgtagacaaagaaccctgcagtgagacccttcaggctccccaccgaaaaccagagataagtagaggcaaaactcatcaactccggcactgaggggaagagactgggggaagggactgtgctgggaagacacttactttactgtggtaaaacttcacgcgttgatggcctgcccggatcggttttgttttggtcagatgaatttcacgagctggacgtgatggacatcagattagttcggttgacgtcagatgttctctgcactcagtattgatttatgaaaggttgacgacactatttttatagtaaagtaacttacagcaataggacaaacgagactgcttggaaattaacaacttctagttgtgtgtttgagtaaaaaatagttttgttttgcaaaatactgatgattttgtagtcattcagagcagaaaatgattatgatattttaaattgttttaaataacaaaaaaaaatgccatgtgtatgttaatttttccaatattttaatttcagataagtcattacagtggaataccctggatttgtagacactttgttattctgaccagttgtcatttagagagagaaaaaaacactgtaaatgaccatatttaatttctcaaattacagaataattttgatattaacattttactcaagcacataaatagaaaaaaaaactgtttgaaaaacatttttccatagctatttttgtgatgtctattaatattttcattgcattcataaaaagaaaaatgaaagccatgcatttcagcctgaaaaaaaacttgtacattgtctgtaaaaaatgaaaatttgtatttattttattaaaatatttaatgcaaaaata

***wnt5b***

MO GTCCTTGGTTCATTCTCACATCCAT

Wnt5b 2’OMe ASO#1 (GC = 40%)

mU*mG*mG*mU*mU*C*A*T*T*C*T*C*A*C*A*mU*mC*mC*mA*mU

Wnt5b 2’OMe ASO#2 (GC = 50%)

mU*mG*mG*mU*mU*G*A*A*C*A*G*C*T*T*G*mU*mC*mC*mG*mU

>NM_130937.1

cctaacaagtgtgcaaagccatctttccctgaagtctttcctattgacggacaagctgttcaaccaataaagcacaggtttttgctcgggatacatctttcttccatttcgacaatcaacttttatccaacttccaagaacttccatctgaggaggcgctttgcatttcaatttcttggaggaggaagg**ATG**gatgtgagaatgaaccaaggacacctacttctggcagtgaccctcatcgtctgcaactcacagctgctggtggtcgccaactcgtggtggtcattagccatgaaccccatccagagaccggagatgtacatcattggagcacagcctctgtgcagccagctgacgggcctatctcagggtcagaggaagctctgccagctctatcaggccacatggtttatattggagagggggcgaagacgggcatcaaagagtgccagtatcagttcagacagaggcgatggaactgcagtacagtggacaacacgtcagtgttcggccgcgtcatgcatataggcagccgagaaacagcttttacgtacgccgtcagcgcagcgggtgttgtgaatgctgtgagtcgagcgtgccgtgagggtgagctttccacctgcggctgcagtcgagcggctcgtcccagagacctgcccagagactggctgtggggcggctgcggggacaacgtcaactatggctaccgcttcgcccgggagtttgtggacgctcgtgaacgtgagaagaactacccacgcggatcggtggaacacgcacgcacgcttatgaatctgcagaacaatgaagccggaagaatggcggtgtataatctagcgaatgtggcctgcaagtgtcatggcgtctcaggctcgtgcagcttgaaaacctgctggctccagctggccgacttccggcgtgttggagaattcctgaaggagaaatacgacagcgccgccgccatgcgcattaaccgacgtggaaaactagagctggtcaataatcgattcaacccaccgacaggtgaagatctggtctacatcgaccccagcccggattactgcctgcgcaatgaaaccactgggtctctgggcacccaaggccgcctatgcaacaagacctcggagggtatggacggctgcgagctcatgtgctgcggccgcgggtacgaccagttcaagacctacaaacatgagcgctgccactgcaagttccactggtgctgctatgtcaagtgcaaacgctgcacgtcactcgtagaccagtttgtgtgcaagtagcagacgtgagaactgggggacagacgcactgagcaattaagcaggagaagaaacgggacccttacggacccagaggggcaacttagagataattaaatgtaaaaatgatatattaaatagcaacaaattaaaagtatataaataagtgtacgtgtgccgttgatagtaatttaatgacctgaggatccacgttggacgtcattgatgaaaaaggaggcaccggtgtgatgcattccggctcaagctgtgtttctcttatatagagaggaaggaactcttgacttgtgctacaagaaactctttagagactcgaggaaaaggcgcagaaataggatggggaacatcaagggcgcatcacccacccattgccttccaattcttaaacacacacacacacacacacactccaccttgctgatgtcagagctcttaagaactttcggaatgcg

***MALAT-1***

MALAT-1 2’OMe ASO#1 (GC = 45%)

mU*mC*mC*mA*mC*A*C*A*A*C*A*T*C*C*T*mG*mG*mA*mA*mU

MALAT-1 2’OMe ASO#2 (GC = 55%): used for knockdown experiments

mA*mG*mC*mA*mU*T*C*A*G*G*G*A*C*A*C*mU*mG*mC*mA*mC

MALAT-1 2’OMe ASO#3 (GC = 30%): also produces knockdown

mU*mG*mA*mT*mA*C*G*G*T*T*T*G*A*T*T*mU*mA*mA*mA*mG

>Zv9_00034242 (MALAT-1; Pauli et al., 2012)

gacgaggcaacaacattgtgcgtcacgacggggtgaggcgctatggaaggcagggaggcttcgttgatctggtgaaatgttcttattttgttctgtttaattttttgataactaataggcatttttatttacaaacctttaaagtcttaaaactctagacgttttccgttggttatacaaaggttttcttttgagtagcaagcgtgacgaagataaaatacaagcttacttttcacttaaataggtaacttagaagtgcctttatgagaaaaaattcattttttgtttatcttaaaaagacaaaaaacgtcttatttagtcgaacatttaacgtttatcttcaggttaatgtttaactagcttacgttaccgaaagcctaaacaaagctcgactgtaaatggctaagcgaacgttatccttttcacttaacctcattttaagcttatttttgtttcagcaagggttttctgaaattacgtgattttatacacttatttttttgacgcttgtttagtttgattctcgacgctagaaagaatttacaaaatggcggtcgcgacgacggattagatttcgttgaagaaaagctgttatgatttctcttttgttttcaagtcagaacaaccaataacagtttatataaacctatagagttatattagaccaacgttatatacagacctacattttattatttatttttctttttacaataccgattagtaacagccttttatggtaacagtaaggacccagggtatctacaaccattttaacttgtaaattttcaaggaaaaactttttgtttctttttttatcttcagaccataacgagtaatagcagccttttatggtagtgaggtcaaacagcaagtcttttttttctatcttgatattacgattagtaagttgacccatttagcaaggatgaacttaaaagcaaccagtttaaattagatggagggtaaacgactacaaggttacagtagattttacagtagaaaaaaaaaattcatttttttgtcatttaggttgactagcaaaaccactaacgaccatttcagtacagcaaggaagagcatatttaagctacagactttcttaaaaaaatatatattttagtatgcctgccataacttgaaaatcaattacaacttatatttcatttaaaaaaaaaaaaaagtattttcttttaatataccgcaagtccattgcaacttaattatgatggcaaacattttaaaaatgtatttctttttattataacagccataacaaatgttacaagtttattatggtagtaaacaaaaaaaatctttttaatatgccagctataatgtaaatgtgttacaacttcattatgaatccaaatcagttttattaaaacaaagcagctgcttttgttaaaaaaaaaaaaaaaacagcagagattaaataaaccgatctacaagactagatttttctaaaatctgaaagaacagtctagaactgccaatgagatagaggattaacacagtcaagactgcagtagattttcttaaaccagttttgtttttacaattttaaccttttaatggtcaggataaaaaaaaaaaacactattactgaagatttcagttttctttttagtttgcatacacttaaggtatagtgagggcaaaccatcattgctatgttgtaaatctcattttattttaaacagtcatttatttatttttttacccgttttatatacagtagtacaaataaataactacaatctaaggctacagtagatcttaaagttattttgcttgtaaactattacacaataataaccacttaagtagcaaaaacagcctactggagatttaaaaaataaaaaaaaaaagaactttttctccaacccaaaacttagcggcttatatgatagtaagggtgaggttgtccacagtagaagtcaacagtagatttttatttattttcttttgggaaagaaaacttttaatttagtatttcagtctcttgactaattatagagggtatcaataattagtcaagattactttttttttttttttaaactacaatagaacagtttacgttaccaagaatgcagtatttacatttttatttgtttatctgcaaaccttatccaagaaatgaggaacagcttctgatagccagagtgccatttttaagagattttattttgttttttgcataccataacagaacaatcaccaacgcttaaagccaaaaaaaaaaagccagacaattttttgaaaactacaatagaacagtttacgttaatcaagaatgcagtatttacatttttatttgtttatctgcaaaccttatccaagaaatgaagaacagcttctgatagccagagtgacatttataagagatttttattttgttttttgcataccataacagaacaatcaccaacgcttaaagccaaaaaaaaaaaaaaaaagccagacaatcagatgtaaaagtctttttaaatattacttttgaaaaaaaaaaaaaaaagcctgctcttgttgcctattaggaagaaattactgaacatcaagtgagtgtttttagtttggaagacaacgattatagatgctgcctcctttttttttctttttttttttttcttttttttttttgagaagaagaagaattgagtatttcaagatttaaatttgcaagcctagaagaatcaatcaacaatgatgctttttttttctactgaaccacgtaattgattgctgtatggactgagtcatgttgagtaacagcagtgaaatggaagcgtaggttgttttcatgatccaaagatatcagcagaagacggatgtgcagaggacaggagaaatggactgagtttatccagtaacaacatagctcttagatgacgtcggatggaagaaaaagatctacagcagggaccagggagaatcaggacaagaaaagatctacagcaggggccaggaccagggagaatcaggacaaggtggcgagccaggcagaaccaggggaggatcagagggcacagatcagggtgaagcgcagcagtatccagaagggggagccgggtctacaaggcagagtagcttcagatccacgttgaccaccagggggagctcagctggtctagatcagagcaagccaaagatgggtgtcccaaaagcagcaggagaaagagggctcacaagcagagggtatcaggggtcgttgccatagcacagtcatgaggagggcatttggtttgtatgggttgaggtattctgtgtaacttaagtcattatttcagtttgtattccagaaccctttgctcgcgaagttctgttctttccttttcaggcgtggtgaatgaatgtttgaaaatccgatatggcatctgtcccgttgttggccaagtctgatgaatgccgttagtgtgctgcggagacgatcgtccttattcgaccacgcctccgctgagatttggctataggcccgtggaaaagtcgtcccccatatccgcttaaatccattaggtctgtcctgagaagcaatccacgttcgaagggtttttcggagttgatgttcgactgcgtccctacacgacaccactacctctactgcaacacccgctgcgtcagtccaacatatgatgccagggcagccgcatattgcctgccggtaaaattttgatgagttcatgcatgaatttttattgtaatttagagcaaaagctttgtatatttaaaaaaaaaaaaaagaaaaaaaaaagaaaagagaaaaaatatatacaatacgtttttgcaaaagtaaatgcgttattgtaaaattattaacatacagatgttgcaaatgcactttctgagtagttatgtaatgttattgcagtgtttttggggtttgggaggggcaaattcgtttctcgagttctctttcccacaggccctgttcacattagaggcgataaaaccaaaaagggattccaggatgttgtgtggacccttttagagtcagtttgacttccctgtaaagaattctttaaaagcttcaaggaaagcctctgatttaggaaggtaccccattggatttgttttaaagtcttgtttggaatcatgaactatacatttatcaatattttccagtattacatgttgggatttgcaatggggtgggaggggtgtgggtcgttaacccaaatcttttcctagactttgcagcttgtgaaagcggctttattaagggtggcaatggcttgaatagcgcagtatggggaaggaaccaccagtgtgtctgccagtggatgcgtggccctgttacaagccttccatggcagctagaccaacgtttacatgggtaacgcggcatgggatcaaacgctttcattttatttaacacatcattatggccattttcctatcattattgaattggtgtttggatattggttgtgtatttggatattgtttatggggtttgtggatctggggggggggattgcctaattttgactcttacagattgaaacaaacccttaaagaacaaagctggtggtccaaccaatatctgctccacttcccccacttgagcaaagcttcttcgctagaggtccagatgcttttaacattttctaatggcaagtaattgtgggtattcatgtaaatggcattcatttgcttttgtctgtcgttgtattgtacttgaatacaatttgatggctaagtgtaagcagtaactgtattagttgcttatttgtctttttaatttgtgtattttgaaaagcggggatgtgtgtaggctttcttttcctccccctctgcatttttcagtgtgcagtgtccctgaatgctttcaaccgaagatgtcatcctaacattgtttgtaaagatttgctttcgcatcaagcaacttcagcctcttttcggtcacgtaaggtcactaagcaagtccatttgtttctaaccttttccatttttaatagtctgttacaacctgtaaaatgtgtggtcgcacacttattacaaattagttttgtaatttgtatttaagtgtacattctgtaactgttcccattttcagttaataaatgcatagcctgttgtgattgaaattttggtatgcatttgtttcttgggggaatgtgtggggctttcttgggtgtttctttatgctttttcaggtatgagtgtaaagcttggcgctttttgtagcttggatgccagggcttgatgtcaactctgtgcaaggtggagaggcagatcagtccagtcctacagcagtgtgcgaaggaggaaaggcaaattgtatatacaaaatttaaggatataccgtaaatttgtagactctttggctttaagcctgtgcttttgatatctgaatcttgcagatttgatgtttttacttgatcattgggtgggtgtaggggtcttgttgctttgccatttggtcttgcagaatcttgcaaaagccttgaagattggcacctatcagaagaagttatgacctgaaatcgcccgcttccactgaacgcctcatcaggccagacagtcgttctatagattcgtaaccttttaaattttctttaactgcccagccacttctctgcgcaactgtgcagtgacaaatgctttacaaatgtatagcagcagtctttatttcagctgtcgcacaactgtgcttcagattccaaatttaaagtggctgggaacggctgcgcaactgtgcagctttatttggcattggctcaacagccgtacataaactggaaaagatgtgtttggggtgggaaaggggatctgcacttttctctctcttctgcagaaatctcatcagccagctgtgtggtgaaagttacatgatgtcgccctgtggagtctgtagttaccaggtttcttgaatctttaacttgatggtagaatcctcatgcaactttttataaacttcagtaacatcaatcgccaatgcttcagaagctacactacctttatgcttcatgcactttaagtttcttttacatcttaactcctcagttgagtcacagttagtattgcaatcggtgtgtggtggaagtgtttgtgtgtagaagctctttgtttcagattacccgtctgtccccctgccttctcgactgcatccgaatgcaaggctgtctgtgagagcagttgtccccggtgaatgtcgtcttcacttgaatgaatggccaatcgaatccccccttctgtgcgcttgagagctttgtccaaaccttggaagcccttcgccgaagtccctctgtgtgtctgcgcgtcttttttttttctgtttatcccccacctccctctccaattcttcattttcttaggtcaatttcatttagggagcaaaattgtcttgtgtccgtctgggagaaccctaaccgcctgatgaaacccatccactcgctctgatcaacctcattcgtcatattttgctgctaatcgtgcatccctctgctgctatctgctgagttaagtacatgtagttgatcttaatcttgtgtctgggtagaagcattcaccctttttatagatatgcatgcacataacactttaaatcaaaccgtatcagtgtgtgtgttggtcgctttggggagggatggggcaacactttctctcttttcaggtacgctgtttgtgattcctctaaaaattaaggctgagccataacatgggagtggatggatgtgcttgcgtcaaagccgtaactagtacctgcattggttttaaagtgttttttttaaaaattgtgttgggattgggaagagggagggggggttgtgggcttgtaaacgtgttgttcttttcattgcagctatagacttggtaaatcagtactctgaagaactgggtgtaaagcgccgctacctggagataagtctatacgtgcacttttgcctcttaagaaattctgagaatagttcggggagtctggggggaaaccgaaaaccctgcgagtatctgtctagttacaggaatggtgattttacaaaggaggctattttgttctggccgtgttggtggaaatccaagacctgggaccgttgtgtggtggtaaatgacatttgtatcacatttggttatgaatctttggggttgggtggggtgggggggttctaaacccttttctgtgtttcagatttttgtttaaataggatcacgtcacctgaatgcaagtgcagccttgaacaagacctaactagtcagatttgatcttcgaagtaaaggcgtgtgaatatgctgcatattgaagacaaaattactacagtaatatcgatgggttttgaagagttcgactggggattttgggggggaaacctgcaatcttttgtgtagtttcatactaaagaaggaccgttcgagacgagagttttgtagagcagcaggtttgaggtttttcttttttcctgggggaacaatctttggattgttctttcaggttttgctttttaacctcctaagaaaaaaagcaaaagaccctggtggaggcactcctgattctcaggacggggttcaactccctgcggcgtctttgctttctaacctctgctcaatcaattctctttaatgtatctaataaaaattgtttgcttcgaaggaagtgtaaatggattgtttatattttaaggtgcatttagctgatgtagagcaagtgctcctcaaattgatactactgctgatggcttaataaaagtttgtaaccagctttgattcaaatcgtgctctggttcgtatacaaaagtcaaatctcttttgtaatcttatacaacatttatctattctctgcctacttgttttaaggtaacattcatgtgagaacatttattggggtttgtttgaatgtccatgggcattgaaaataatgggaaattgataaagggttcgtttctcagaacgataacctgttacaatgtctatggccagtgaggttatctatttgcatgaactgtaaagtggcaagatctatttatcattttgtatagcagtatcaaataaattaaattaaaagacctgggggtttttttcccccaacacttaagcattggcttttttttatttttatttattttttatgatttgctcataccaagtttccttctgtcagccttcagaaagtgtcattcattatattgcactacttttcatacgttacgtatttatatgcaaaaattaatttgaa

***miR-126***

MO1 TGCATTATTACTCACGGTACGAGTTTGAGTC

MO2 GCCTAGCGCGTACCAAAAGTAATAA

miR126a 2’OMe ASO#1 (GC = 50%)

mC*mU*mC*mA*mC*G*G*T*A*/iMe-dC/*G*A*G*T*T*mU*mG*mA*mG*mU

miR126a 2’OMe ASO#2 (GC = 55%)

mG*mC*mC*mU*mA*G*/iMe-dC/*G*/iMe-dC/*G*T*A*C*C*A*mA*mA*mA*mG*mU

>NR_030071.1

gagccattttaactgcttcacagtccattattacttttggtacgcgctaggccagactcaaactcgtaccgtgagtaataatgcactgtggcagtgggttt
